# Co-fermentation of *Rosa roxburghii* Tratt pulp by *Saccharomyces cerevisiae* and *Bacillus subtilis* alleviates diarrhea-predominant irritable bowel syndrome in mice by reshaping gut microbiota and host metabolism

**DOI:** 10.3389/fnut.2026.1854409

**Published:** 2026-06-05

**Authors:** Jian Zhang, Shuo Zhang, Zhiyu Chen, Chencen Lai, Shiyu Zhang, Fangling Feng, Pengjiao Wang, Xuncai Huang, Min Zhang, Xiuli Gao

**Affiliations:** 1 State Key Laboratory of Discovery and Utilization of Functional Components in Traditional Chinese Medicine and School of Pharmaceutical Sciences, Guizhou Medical University, Guiyang, China; 2 Engineering Research Center of Microbiology and Biochemical Pharmaceutical, Guizhou Provincial Department of Education, Guiyang, China; 3 Experimental Animal Center of Guizhou Medical University, Guiyang, China; 4 Guizhou Provincial Engineering Research Center of Food Nutrition and Health, Guizhou Medical University, Guiyang, China

**Keywords:** co-fermentation, gut microbiota, gut- brain axis, IBS-D, metabolomics, *Rosa roxburghii* Tratt

## Abstract

**Background:**

Diarrhea-predominant irritable bowel syndrome (IBS-D) is a common functional bowel disorder closely associated with gut microbiota dysbiosis and gut-brain axis dysfunction. However, effective treatment options remain limited. In this study, we investigated the therapeutic effects and underlying mechanisms of an extract derived from the pulp of *Rosa roxburghii* Tratt after co-fermentation with *Saccharomyces cerevisiae* and *Bacillus subtilis* (referred to as FRRTP) in a mouse model of IBS-D.

**Methods:**

*Rosa roxburghii* Tratt pulp Co-fermented with *Saccharomyces cerevisiae* and *Bacillus subtilis*, followed by extraction, was analyzed by UPLC-Q-Exactive Orbitrap-rbitrap OrExactive Oron, was ouse model of IBS-D. This study, we investigated the therapeutic effects and underlying mechanisms of an extract derived from the pulp of t version, please let us know that does nointragastric *Senna Folium* extract. After FRRTP intervention, diarrhea symptoms, abdominal withdrawal reflex, behavioral tests, colon and brain histopathology, inflammatory cytokines, and gut-brain neurotransmitters were evaluated. Gut microbiota was analyzed by 16S rRNA sequencing. Untargeted metabolomics was performed on serum and colon samples, and AFADESI-MSI spatial metabolomics was used to map metabolic alterations in the brain.

**Results:**

Co-fermentation significantly increased the variety and content of bioactive components (e.g., flavonoids and polyphenols) in *Rosa roxburghii* Tratt pulp. Compared with the model group, FRRTP alleviated diarrhea, visceral hypersensitivity, colonic damage, and brain histopathological damage, reduced TNF-α, IL-6, and IL-1β levels; modulated brain-gut neuropeptides (5-HT, SP, VIP); and improved anxiety-like behaviors. 16S rRNA sequencing revealed that FRRTP reversed gut microbiota dysbiosis by increasing beneficial bacteria and reducing the proportion of pathogenic bacteria. Metabolomics showed that FRRTP regulated tryptophan, purine, and glycerophospholipid pathways, spatial metabolomics confirmed that FRRTP ameliorated the disturbed cerebral distribution of 5-HT, tryptophan, glycerophosphocholine, dopamine, and adrenaline.

**Conclusion:**

Our findings demonstrate that FRRTP alleviates IBS-D symptoms in mice by modulating the gut microbiota and correcting metabolic disturbances, supporting its potential development as a functional food or therapeutic agent for IBS-D.

## 1 Introduction

Irritable Bowel Syndrome (IBS) is a common functional gastrointestinal disorder characterized by persistent abdominal pain or discomfort, often triggered by multiple factors leading to abnormal gut-brain interactions. Its core clinical presentation consists of abdominal pain and abnormal bowel habits, encompassing alterations in defecation frequency and stool form (1–3). The current global prevalence of IBS ranges from 12.2 to 16.1% (4), with global prevalence increasing annually, making it a significant public health concern. Based on bowel habit predominance, IBS is categorized into four subtypes: IBS-D, IBS-C, IBS-M, and IBS-U. Of these, IBS-D is the most frequently diagnosed, accounting for about 40–60% of all IBS patients (4–6). It is clinically characterized by recurrent abdominal pain accompanied by increased stool frequency and altered stool consistency (mushy or watery stools).

The etiology and pathogenesis of diarrhea-predominant irritable bowel syndrome (IBS-D) remain unclear, involving a complex and multifactorial mechanisms. Research suggests it results from the interplay of multiple factors, including visceral hypersensitivity, gastrointestinal dysfunction, gut microbiota dysbiosis, and central nervous system dysregulation (7). Modern medical management primarily focuses on symptomatic relief due to a lack of targeted drugs or curative approaches. Current clinical strategies mainly involve antispasmodics, antibiotics, prokinetics, probiotics, and psychotropic medications, often combined with dietary modifications and psychological interventions (8, 9). However, therapeutic outcomes are often unsatisfactory. Given its high prevalence and recurrent nature, IBS-D significantly impairs patients’ quality of life. IBS patients frequently experience comorbid psychiatric conditions such as anxiety, and dysregulation of the gut-brain axis plays a pivotal role in the intestinal dysfunction and visceral hypersensitivity associated with IBS-D. Therefore, developing effective treatments constitutes a pressing clinical challenge.

In the intricate pathophysiological network of IBS-D, the gut-brain axis is considered a central bridge linking the central nervous system to intestinal function. Dysregulation of key neurotransmitters along this axis, such as serotonin (5-HT), substance P (SP), and vasoactive intestinal peptide (VIP), can lead to disturbances in intestinal motility, secretory function, and visceral hypersensitivity. Concurrently, the gut microbiota interacts with the host via its metabolites, further influencing gut-brain axis function and establishing a vicious cycle (10). Studies indicate that IBS-D patients often exhibit reduced gut microbial diversity and an altered *Firmicutes/Bacteroidota* ratio (11). Clinical management of IBS-D is currently predominantly symptomatic, often yielding suboptimal outcomes with a high tendency for recurrence. Therefore, developing multi-targeted therapeutic strategies that address the gut microbiota, host metabolites, and the gut-brain axis holds considerable scientific and clinical significance for IBS-D.

*Rosa roxburghii* Tratt, a plant of the genus *Rosa* in the Rosaceae family (12), is primarily distributed in southwestern China, especially in Guizhou Province. Recognized as both a medicinal food and a traditional remedy in ethnic minority regions, it is recorded in the Compendium of Materia Medica (Ben Cao Gang Mu) (13). This classic text documents its functions of fortifying the stomach, promoting digestion, and relieving diarrhea, indicating its use for dyspepsia, abdominal distension, enteritis, and diarrhea (14). The fruit of *Rosa roxburghii* Tratt is rich in various bioactive compounds, including vitamin C, superoxide dismutase (SOD), flavonoids, and polyphenols (15, 16). In recent years, growing research has progressively validated its medicinal and health-promoting values. Current studies have demonstrated that *Rosa roxburghii* Tratt possesses hypoglycemic (17), anti-aging (18), antitumor (19), and anti-stress activities (20). However, its potential effect on diarrhea-predominant irritable bowel syndrome (IBS-D) has not been investigated.

The juice processing of *Rosa roxburghii* Tratt fruit generates a significant by-product, accounting for approximately 50% of the total fruit weight. This residual pulp is often discarded as waste, leading to considerable resource wastage and environmental concerns (21). In fact, the pulp is a valuable resource for reuse, comprising more than 70% dietary fiber while retaining a considerable proportion of bioactive components such as polyphenols and flavonoids (22).

Microbial fermentation technology is an effective strategy for enhancing the bioactivity of by-products from traditional Chinese medicine and agricultural processing (23). In particular, the co-fermentation system employing*Saccharomyces cerevisiae* and *Bacillus subtilis* can degrade plant cell walls, thereby facilitating the release and transformation of active constituents. Furthermore, the fermentation process may generate novel bioactive compounds and improve the flavor and bioavailability of the raw material. Applying this technology to *Rosa roxburghii* Tratt pulp is a promising approach to developing new functional products aimed at modulating the gut microbiome and alleviating IBS-D symptoms.

Currently, although a few studies have explored the gut-modulating functions of dietary fiber derived from *Rosa roxburghii* Tratt, research has predominantly focused on its laxative effects. There is a lack of systematic investigation into its intervention effects on IBS-D and the underlying mechanisms, particularly after co-fermentation with specific microorganisms such as *Saccharomyces cerevisiae* and *Bacillus subtilis.*

Therefore, this study aimed to process *Rosa roxburghii* Tratt pulp through co-fermentation with *Saccharomyces cerevisiae* and *Bacillus subtilis*, followed by ultrasonic extraction. We first analyzed the alterations in the material basis and the content of major active components before and after fermentation. Subsequently, we systematically evaluated the effects of the fermented extract in a *Senna Folium* aqueous extract-induced mouse model of IBS-D. The assessment encompassed intestinal symptoms, neurotransmitters, inflammatory factors, behavior, histopathology, gut microbiota structure, and the host metabolome. Ultimately, this work seeks to elucidate the underlying mechanisms from an integrated perspective of the gut microbiota, metabolomics, and the gut-brain axis, aiming to provide novel insights and experimental evidence to support the high-value utilization of *Rosa roxburghii* Tratt pulp and the development of dietary interventions for IBS-D.

## 2 Materials and methods

### 2.1 Materials, reagents, and animals

#### 2.1.1 Materials and reagents

*Rosa roxburghii* Tratt pulp, The pulp of *Rosa roxburghii* Tratt was harvested from Qiannan, Guizhou, and authenticated by Professor Qing-de Long at Guizhou Medical University; *Sennae Folium*, purchased from Kangmei (Bozhou) Century Chinese Medicine Co., Ltd. (Bozhou, China); Rutin, Ursolic Acid, Gallic Acid, Ellagic Acid purchased from Beijing Solarbio Technology Co., Ltd. (Beijing, China); Luteolin, Glucose, Kaempferol, Catechin, Quercetin, Flavone purchased from Shanghai Yuanye Biotechnology Co., Ltd. (Shanghai, China); 5-HT, SP, VIP ELISA Test Kits purchased from Shanghai Kexing Trading Co., Ltd. (Shanghai, China); TNF-α, IL-6, and IL-1β ELISA kits were purchased from Quanzhou Ruixin Biotechnology Co., Ltd. (Quanzhou, China); High-activity dried brewer’s yeast: purchased from Angel Yeast Co., Ltd. (Yichang, China); *Bacillus subtilis* was isolated from Guizhou specialty Douchi.

#### 2.1.2 Experimental animals

Male C57BL/6J mice, specifically pathogen-free (SPF) and between the ages of 6 to 8 weeks, were acquired from SPF (Beijing) Biotechnology Co., Ltd. (Animal Production License No.: SCXK (Jing) 2024-0001). Mice were housed at the Laboratory Animal Center of Guizhou Medical University under controlled conditions: 22 ± 2°C, 40–60% relative humidity, and a 12-h light/dark cycle, with *ad libitum* access to food and water. This study protocol was approved by the Animal Care and Welfare Committee of Guizhou Medical University (Approval No.: 2400722). To exclude potential sex-based differences, only male mice were utilized in this study.

### 2.2 Methods

#### 2.2.1 Preparation of *Sennae Folium* aqueous extract

One hundred grams of *Sennae Folium* were weighed and soaked in 1 L of ultrapure water for 30 min. The mixture was brought to a boil over high heat and then simmered for 10 min. After filtration, the filtrate was collected and concentrated under reduced pressure to a final volume of 100 mL using a rotary evaporator at 60°C, yielding a decoction with a concentration of 1 g/mL (equivalent to crude drug). The extract was stored at 4°C for subsequent use.

#### 2.2.2 Microbial culture conditions

Activation of *Saccharomyces cerevisiae*: An appropriate amount of *Saccharomyces cerevisiae* was weighed and mixed with warm water at a ratio of 1:20 (m/v). Sucrose was then added at 5% (w/w) of the yeast weight. The mixture was incubated for activation in a constant temperature shaker at 37°C for 30 min.

Culture of *Bacillus subtilis*: The activated *Bacillus subtilis* was inoculated into a nutrient broth medium. The culture was incubated in a thermostatic shaker set to 37°C and 180 rpm for 18 h.

#### 2.2.3 Preparation of *Rosa roxburghi*i Tratt pulp extracts

The pulp was obtained by juicing fresh *Rosa roxburghii* Tratt fruits, followed by drying and storage. The dried pulp was then subjected to co-fermentation using *Saccharomyces cerevisiae* and *Bacillus subtilis*. Specifically, the pulp was mixed with distilled water at a ratio of 1:2 (m/v). According to the optimal fermentation conditions established in our previous study (24), *Saccharomyces cerevisiae* and *Bacillus subtilis* were inoculated into the mixture at a ratio of 1:3, with a total inoculation volume of 4%. Fermentation was conducted at 31°C for 24 h in a constant temperature incubator; the product was then oven-dried at 50°C.

Both the unfermented and fermented pulp samples were subsequently extracted using an ultrasonic-assisted method. Extraction was performed three times (1 h each) with a solvent system of ultrapure water and 80% ethanol, under the following conditions: Extraction was performed under the conditions of a 1:10 solid-to-liquid ratio, 400W power, and 50°C. The combined filtrates were then concentrated under reduced pressure to an appropriate concentration, and finally lyophilized to obtain the *Rosa roxburghii* Tratt pulp extracts.

#### 2.2.4 UHPLC-ESI-Q-exactive plus orbitrap-MS analysis

To identify potential bioactive chemical compounds in the fruit pulp extract of *Rosa roxburghii* Tratt, we employed a Waters Ultra Performance Liquid Chromatography (UPLC) HSS T3 column (dimensions: 2.1 × 100 mm, particle size: 1.8 μm; manufactured by Waters Corporation, United States). The mobile phase comprised (A) 0.1% formic acid dissolved in acetonitrile and (B) 0.1% formic acid in aqueous solution. The following chromatographic conditions were applied: injection volume, 2 μL; flow rate, 0.3 mL/min; and column temperature, 40°C. The gradient elution procedure was implemented as follows: from 0 to 2 min at 5% A (isocratic); transitioning from 2 to 42 min from 5 to 95% A (linear gradient); from 42 to 47 min at 95% A (isocratic); from 47 to 47.1 min decreasing from 95 to 5% A (linear gradient); and finally, from 47.1 to 50 min at 5% A (for re-equilibration). Mass spectrometric analysis utilized a Thermo Scientific Q Exactive Plus hybrid quadrupole-Orbitrap instrument (Thermo Fisher Scientific, United States). For the specific instrument conditions we referred to earlier research conducted by our group (25).

Data analysis was conducted utilizing Xcalibur software (version 14.1, Thermo). The elemental compositions and molecular structures of the chromatographic peaks were deduced from high-resolution mass spectrometry data, allowing for a mass error tolerance of 5 ppm. Compound Discoverer 3.3 (Thermo) equipped with the mzCloud and ChemSpider databases was utilized for auxiliary compound identification.

#### 2.2.5 Quantification of bioactive components in *Rosa roxburghii* Tratt pulp extracts

The contents of total flavonoids, total polyphenols, total triterpenoids, and total polysaccharides in the different extracts were quantified by UV-Vis spectrophotometry. The concentrations of specific compounds—including Ellagic acid, Catechin, Luteolin, Quercetin, kaempferol, rutin, and Gallic acid—were determined by HPLC. Detailed methodologies are provided in Supplementary material 1.

#### 2.2.6 Animal model establishment and grouping

C57BL/6J mice were randomly allocated to six groups (*n* = 8): the Normal Control group (Control), the Model group (Model), the unfermented Alcohol Extract group (RRTR, 0.4 mg/g), and three Fermented *Rosa roxburghii* Tratt Pulp Alcohol Extract groups at low (0.2 mg/g), medium (0.4 mg/g), and high (0.8 mg/g) doses, designated as FRRTP-L, FRRTP-M, and FRRTP-H, respectively. An IBS-D mouse model was established by combining combined restraint stress, Tail-pinching stress, and *Senna Folium* aqueous extract administration. Specifically, during the 10-day modeling period, mice in the model groups received a daily morning gavage of the extract at 0.1 mL per 10 g body weight. This was followed by 2 h of restraint stress, implemented by securing all four limbs in 50 mL conical tubes. In the afternoon, a 1-h Tail-pinching stress was applied to the distal one-third of the tail. Mice in the Normal Control group were not subjected to any of these procedures. Following the modeling phase, mice in the respective groups were administered their corresponding extracts via oral gavage for a 2-week intervention period. Throughout the study, the general condition of the mice was monitored. Body weight and the diarrhea index (calculated as the sum of the fecal consistency score and the loose stool rate) were recorded daily.

#### 2.2.7 Body weight measurement

Body weight was monitored on a daily basis across the modeling and intervention phases for all mice.

#### 2.2.8 Bristol stool form assessment

On the first day after the completion of drug administration, each mouse was housed separately. Fecal samples were obtained hourly during a 6-h timeframe. The consistency of the stool from all samples was evaluated according to the Bristol Stool Form Scale, and an average score was determined and documented for every mouse. The criteria for scoring are detailed in Table 1.

**TABLE 1:** Bristol stool form scale.

Bowel status	Score	Description
Constipation	1	Passes hard, pellet-like stool with difficulty
	2	Sausage-shaped stool consisting of lumps
Normal	3	Sausage-shaped but fissured stool
	4	Well-formed, smooth, and soft sausage-like stool
	5	Mushy stool with well-defined edges that passes easily
Diarrhea	6	Mushy or pasty stool that is poorly formed
	7	Liquid stool with no solid pieces

#### 2.2.9 Determination of fecal water content

Four hours following the final administration, mice from each group were placed individually in metabolic cages to facilitate the separate collection of urine and feces. Fecal samples were gathered from each cage and weighed to determine the wet weight. These samples were then dried overnight in an oven set to 65°C and reweighed to establish the dry weight. Fecal water content (%) was calculated using the formula: [(Wet Weight − Dry Weight)/Wet Weight] × 100.

#### 2.2.10 Assessment of visceral pain sensitivity via abdominal withdrawal reflex (AWR)

Animals were fasted for 12 h post-dosing with free access to water. For colorectal distension, a lubricated 6Fr double-lumen catheter (paraffin oil) was inserted into the colon, advancing the balloon tip to about 1 cm from the anus. Each mouse was then placed in a restraint device that prevented forward or backward movement. After a brief acclimatization period to ensure the animal was calm, graded colorectal distension was performed. The balloon was inflated with air, starting at a volume of 0.25 mL. The volume was incrementally increased to 0.35 mL and then to 0.50 mL at 5-min intervals. Each target volume was maintained for 1 min, during which the behavioral response of the abdominal wall to the distension was observed. The detailed scoring criteria are presented in Table 2.

**TABLE 2:** AWR Scoring System (Abdominal Withdrawal Reflex).

Score	Description
0	No behavioral response to colorectal distension (The mouse remains calm)
1	Brief head movement in response to colorectal distension; body otherwise immobile
2	Abdominal contraction that does not result in lifting the abdomen from the platform
3	Abdominal contraction strong enough to lift the abdomen from the platform
4	Arched body posture with pelvic structure elevated; severe abdominal contraction

#### 2.2.11 Forced swimming test (FST)

The FST was conducted in a transparent cylinder (12 cm diameter) containing 25 cm deep purified water at 23–25°C, into which mice were individually placed. Each mouse underwent a single 6-min test session, and its behavior was recorded. The duration of immobility during the final 4 min of the session was analyzed using video tracking software (26).

#### 2.2.12 Open field test (OFT)

The test was conducted in a quiet, evenly illuminated square arena (40 × 40 × 40 cm) without internal shadows. Mice were gently placed in a fixed corner and permitted to explore the arena freely for 5 min. Its behavior was recorded by an overhead camera, and movement trajectories were analyzed using the ANY-maze video tracking system. The center zone was defined as the central 25% area (20 × 20 cm) of the arena floor. Parameters analyzed included the number of entries into the center area, total distance moved, and total immobility time in the open field (27).

#### 2.2.13 Sample collection and processing

Upon completion of the intervention period, the mice were euthanized by intraperitoneal injection of sodium pentobarbital at a dose of 150 mg/kg body weight. Blood samples were immediately collected, and then colon, brain, thymus tissues, as well as feces, were gathered. Sections of the colon and brain tissues were preserved in 4% paraformaldehyde for later histopathological evaluation. The remaining samples were quickly frozen and kept at –80°C for additional analyses.

#### 2.2.14 Organ coefficient determination

The thymus index (expressed in mg/g) was derived from the ratio of thymus weight (mg) to final body weight (g).

#### 2.2.15 Histological staining: hematoxylin-eosin (H&E) and Nissl staining

H&E Staining: Colon and brain tissues were fixed in 4% paraformaldehyde, followed by paraffin embedding and sectioning. The prepared sections underwent dewaxing, rehydration through a series of ethanol concentrations, staining with hematoxylin, differentiation, bluing, counterstaining with eosin, dehydration, clearing with xylene, and were ultimately mounted in neutral resin for visualization under a light microscope.

Nissl Staining: Entire mouse brains were fixed using 4% paraformaldehyde and then processed into paraffin sections. Following dewaxing, these sections were subjected to staining with Cresyl Violet (the Nissl stain), rinsed in distilled water, differentiated, dehydrated through an absolute ethanol series, cleared in xylene, and mounted with neutral resin for subsequent observation under an upright light microscope.

#### 2.2.16 ELISA for cytokine and neurotransmitter quantification

Levels of interleukin-6 (IL-6), tumor necrosis factor-alpha (TNF-α), interleukin-1 beta (IL-1β), serotonin (5-HT), substance P (SP), and vasoactive intestinal peptide (VIP) in serum and colon tissue samples were measured using commercial ELISA kits, strictly following the manufacturers’ protocols.

#### 2.2.17 Gut microbiota 16S rRNA gene sequencing and analysis

Fecal specimens were employed for genomic DNA extraction in accordance with the guidelines outlined by a commercial DNA extraction kit. The evaluation of DNA integrity and purity was carried out using 1% agarose gel electrophoresis, and the concentration and purity were assessed with a NanoDrop One spectrophotometer. Following this, the ensuing procedures were implemented based on suggestions from previous studies (25).

#### 2.2.18 Serum and colon tissue sample preparation

Sample preparation followed a previously described protocol. For serum, 100 μL was mixed with 300 μL of acetonitrile-methanol (1:1, v/v). Following thorough vortexing, the mixture was incubated at −20°C for 30 min and centrifuged (14,000 rpm, 15 min, 4°C), with the supernatant collected. For colon tissue (∼100 mg), homogenization was performed in 0.5 mL of pre-cooled methanol-acetonitrile-water (2:2:1, v/v/v). The homogenate was vortexed, sonicated in an ice bath for 15 min, and kept at −20°C overnight. After centrifugation (15,000 rpm, 15 min, 4°C), the supernatant was collected.

Finally, the supernatants from both serum and colon tissue preparations were filtered through a 0.22 μm membrane filter. The filtrates were transferred into vials for subsequent metabolomic analysis.

#### 2.2.19 Untargeted metabolomics analysis

We performed untargeted metabolomic profiling on mouse serum and colon samples with a UHPLC-HESI-Q Exactive Plus Orbitrap MS system (Thermo). A Thermo Fisher Hypersil GOLD column (2.1 × 100 mm, 1.8 μm) was employed for chromatographic separation, for the specific instrument conditions we referred to earlier research conducted by our group (25).

The raw data from the mass spectrometry analysis underwent processing through CD 3.3 (Compound Discoverer 3.3) software for tasks such as peak picking, alignment, and correction of retention time. To differentiate metabolic profiles across groups, using SIMCA 14.1 (Umetrics, Sweden), OPLS-DA was performed to identify differential metabolites, which were defined by a variable importance in projection (VIP) score greater than 1.0 combined with a *p*-value below 0.05. Metabolite identification was achieved by referencing databases like HMDB, MassBank, mzCloud, and KEGG. For pathway analysis, the MetaboAnalyst 6.0 platform was employed, Metabolic pathways were deemed significantly altered if their impact value exceeded 0.1.

#### 2.2.20 AFADESI-MSI spatial imaging analysis

Following collection, mouse brain tissue samples were immediately flash-frozen at –80°C for subsequent analysis. Prior to sectioning, the tissues were transferred from –80 to –20°C and thawed for 24 h. Serial coronal sections were prepared using a Leica CM3050S cryostat (Leica Microsystems, Wetzlar, Germany). The frozen tissue was mounted on the cryostat stage and sectioned at a thickness of 12 μm with the chamber temperature set at –20°C. The tissue sections were dried in a vacuum desiccator for 90 min. Regions of interest were delineated. Spatial imaging analysis was then performed on the sections using an AFADESI-MSI platform coupled to a Q-OT-qIT hybrid mass spectrometer (Thermo, San Jose, CA, United States) operating in positive ionization mode. The specific instrumental parameters and scanning methods followed those described in our previous study (28).

#### 2.2.21 Statistical analysis

All data are presented as the mean ± standard deviation (SD). For two-group comparisons, Student’s *t*-test was used. Statistical significance for multi-group comparisons was assessed by one-way ANOVA followed by Tukey’s *post-hoc* test, using SPSS (version 22.0; IBM Corp.). A *p*-value of less than 0.05 was deemed significant.

## 3 Results

### 3.1 Tentative identification of compounds in *Rosa roxburghii* Tratt pulp extracts before and after fermentation

**FIGURE 1:**
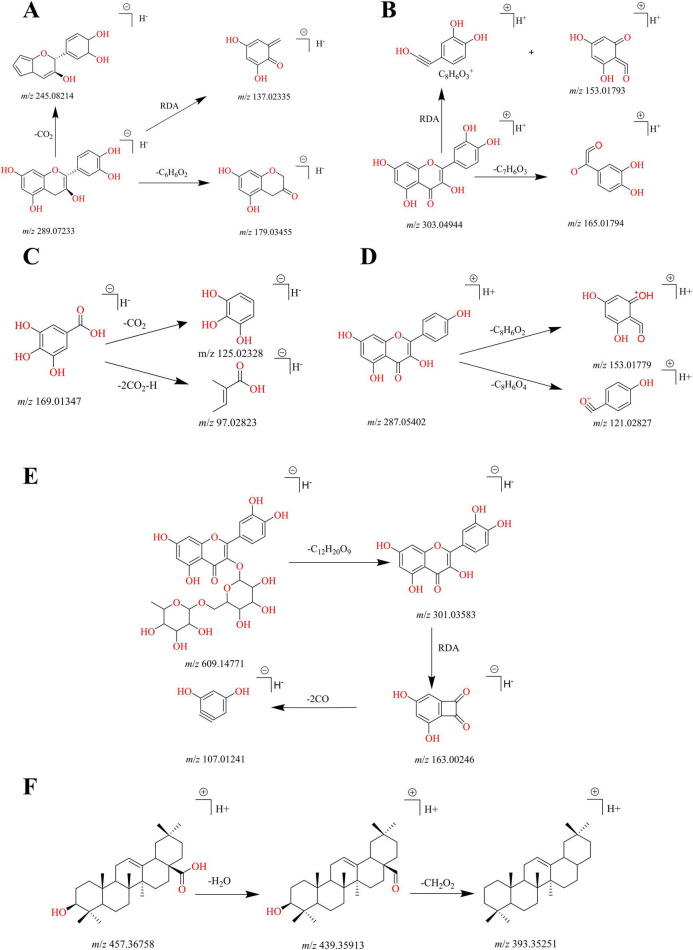
Representative MS/MS fragmentation spectra supporting compound identification. Structures and fragmentation pathways are shown for **(A)** Catechin, **(B)** Quercetin, **(C)** Gallic acid, **(D)** Kaempferol, **(E)** Rutin, and **(F)** Oleanolic acid.

Compounds in the fermented and unfermented *Rosa roxburghii* Tratt pulp extracts were tentatively identified using a UHPLC-ESI-Q-Exactive Plus Orbitrap MS system. Compound annotation was performed by integrating data processing with Compound Discoverer 3.3 and Xcalibur 14.1 software, consulting relevant literature, and matching the observed accurate molecular masses with MS/MS fragmentation patterns. Representative fragmentation pathways are illustrated in Figure 1. Fermentation increased the number of tentatively identified compounds: from 31 to 43 in aqueous extracts, and from 40 to 51 in 80% ethanolic extracts. These results suggest that fermentation increased the number of detectable compounds, and that ethanolic extraction yielded a greater number of compounds than aqueous extraction. Consequently, the ethanolic extract was selected for all subsequent animal experiments (detailed results are provided in Supplementary material 2).

### 3.2 Determination of compound content in *Rosa roxburghii* Tratt pulp extracts before and after fermentation

**FIGURE 2:**
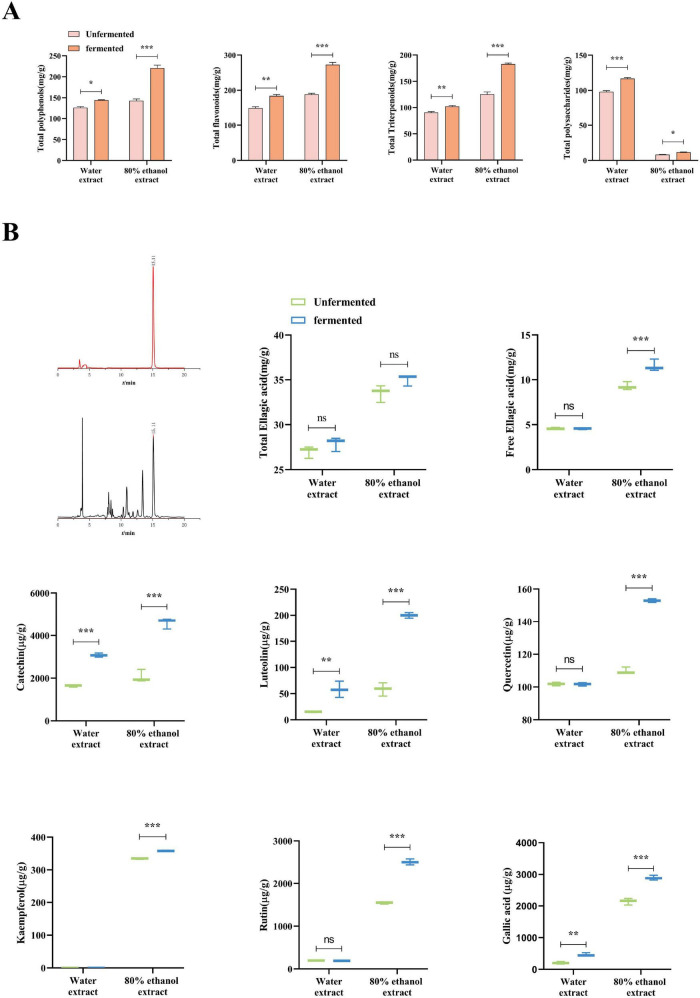
Bioactive compound contents. **(A)** Total polyphenols, flavonoids, triterpenoids, polysaccharides. **(B)** HPLC analysis: representative chromatogram (ellagic acid) and contents of ellagic acid (total/free), catechin, luteolin, quercetin, kaempferol, rutin, gallic acid. (In this figure, ns indicates no statistically significant difference, and the asterisks denote significance levels compared to the value before fermentation: *P < 0.05, **P < 0.01, ***P < 0.001.)

Compared to the unfermented extracts, the fermented aqueous and 80% ethanolic extracts showed a marked increase in the content of several characteristic compounds. This suggests that the fermentation process promoted biotransformation and may have enhanced the bioactivity of the extracts. The quantitative results are presented in Tables 3, 4 and Figure 2.

**TABLE 3:** The components analysis in *Rosa roxburghii* Tratt pulp extract.

Compound class	Water extract		80% ethanol extract	
	Unfermented	Fermented	Unfermented	Fermented
Total polyphenols mg/g	126.40 ± 2.72	144.69 ± 0.69	143.05 ± 4.17	220.70 ± 6.98
Total flavonoids mg/g	148.99 ± 4.07	184.13 ± 4.10	188.31 ± 3.12	272.74 ± 6.59
Total triterpenoids mg/g	90.49 ± 1.77	102.36 ± 1.69	125.47 ± 4.42	182.93 ± 1.89
Total polysaccharides mg/g	98.12 ± 1.55	116.85 ± 1.52	8.36 ± 0.24	11.68 ± 0.38

Data are presented as the mean ± SD (*n* = 3).

**TABLE 4:** Determination results of the monomer component content of *Rosa roxburghii* Tratt pulp extract.

Individual compound	Water extract		80% ethanol extract	
	Unfermented	Fermented	Unfermented	Fermented
Total ellagic acid mg/g	27.00 ± 0.66	27.90 ± 0.78	33.52 ± 0.95	35.02 ± 0.61
Free ellagic acid mg/g	4.59 ± 0.087	4.56 ± 0.091	9.28 ± 0.45	11.55 ± 0.68
Catechin μg/g	1637.10 ± 51.73	3076.88 ± 97.97	2071.87 ± 279.24	4593.54 ± 252.04
Luteolin μg/g	15.34 ± 1.41	57.94 ± 15.50	58.55 ± 12.84	199.92 ± 5.47
Quercetin μg/g	101.86 ± 1.06	101.73 ± 1.05	109.92 ± 1.98	152.90 ± 1.14
Kaempferol μg/g	–	–	334.61 ± 2.22	358.35 ± 1.71
Rutin μg/g	197.37 ± 5.08	186.90 ± 4.30	1545.43 ± 25.98	2506.42 ± 70.99
Gallic acid μg/g	204.75 ± 39.24	465.07 ± 52.99	2141.49 ± 105.17	2889.59 ± 80.03

Data are presented as the mean ± SD (*n* = 3).

### 3.3 FRRTP ameliorates anxiety and diarrhea in IBS-D model

#### 3.3.1 FRRTP improves diarrhea symptoms in IBS-D mice

**FIGURE 3:**
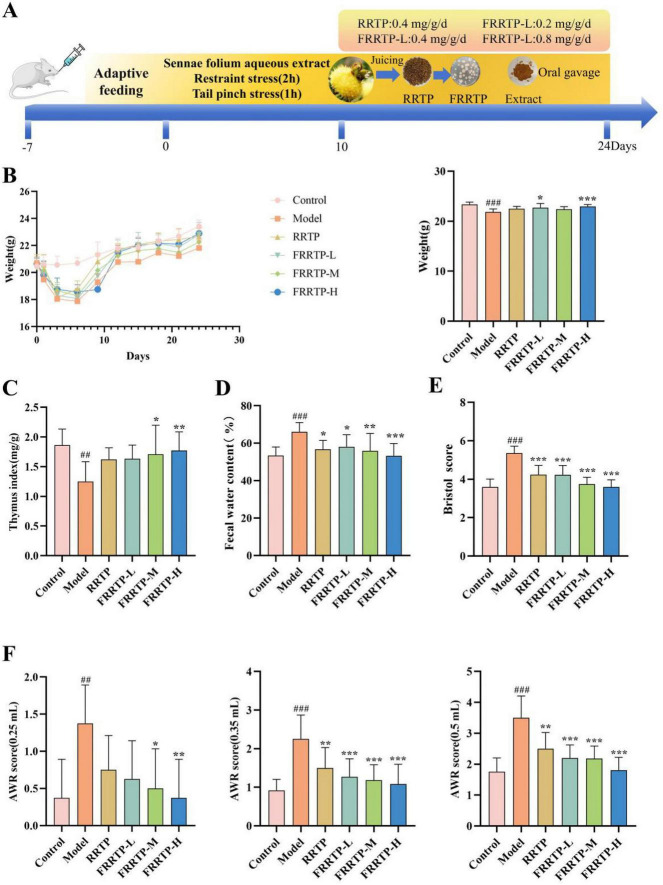
Therapeutic effects of FRRTP on IBS-D mice. **(A)** Schematic diagram of the experimental timeline and FRRTP administration in IBS-D mice. **(B)** Body weight changes of mice in each group during the experimental period. **(C)** Thymus index of mice. **(D)** Fecal water content of mice. **(E)** Bristol stool form scores of mice. **(F)** Abdominal withdrawal reflex (AWR) scores at colorectal distension volumes of 0.25, 0.35, and 0.50 mL. Data are presented as the mean ± SD. (**^##^***P* < 0.01, **^###^***P* < 0.001 vs. Control group; *P < 0.05, **P < 0.01, ***P < 0.001, vs. Model group).

To assess the impact of FRRTP on IBS-D symptoms, an IBS-D mouse model was established (Figure 3A). Control group mice appeared healthy, as indicated by normal feeding, active behavior, and glossy fur. In contrast, mice in the Model group appeared lethargic, showed increased resistance during gavage, had dull and unkempt fur, often huddled together, and their cages required more frequent cleaning due to loose, watery stools. Behaviors such as arching of the back, abdominal lifting, and excessive licking of the abdomen were frequently observed in the Model group.

**FIGURE 4:**
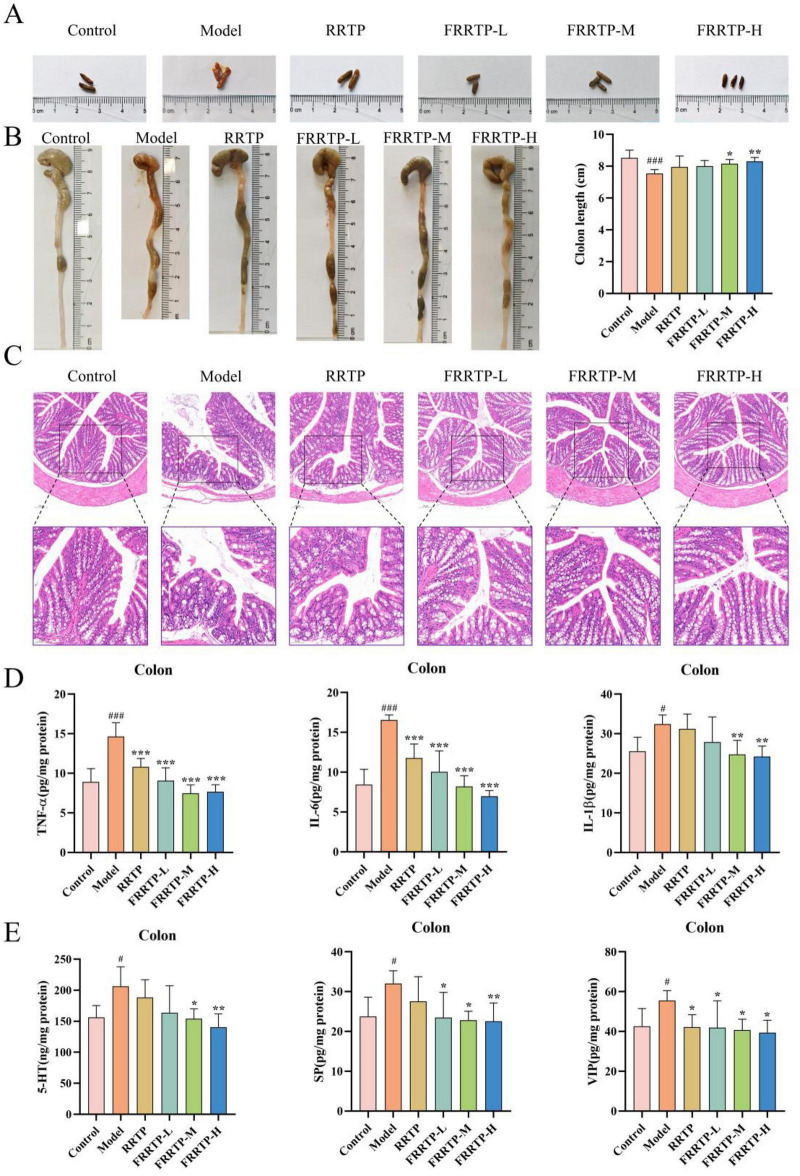
FRRTP ameliorates colon injury and neurotransmitter dysregulation in IBS-D mice. **(A)** Representative stool form from each group of mice. **(B)** Colon length of mice in each group. **(C)** Representative colon pictures, H&E staining [scale bar equals 100 μm (top) and 50 μm (bottom)]. **(D)** Colon levels of TNF-α, IL-6, and L-1β. **(E)** Colon levels of 5-HT, SP, and VIP. (**^#^***P* < 0.05, **^###^***P* < 0.001 vs. Control group; *P < 0.05, **P < 0.01, ***P < 0.001, vs. Model group).

Figure 3B shows that body weight gain was delayed and final weight was significantly reduced in the Model group relative to the Control (*P* < 0.05). FRRTP-L and FRRTP-H treatment significantly increased body weight compared with the Model group (*P* < 0.05). Additionally, the thymus index was significantly lower in the Model group, and this reduction was significantly reversed by FRRTP-M and FRRTP-H (*P* < 0.05), suggesting that FRRTP ameliorated thymus atrophy and functional impairment in IBS-D mice (Figure 3C). The Model group exhibited increased Bristol stool scores and fecal water content compared with the Control group. These changes, indicative of diarrhea, were both attenuated by administration of varying doses of FRRTP (*P* < 0.05, Figures 3D,E, 4A).

#### 3.3.2 FRRTP reduces visceral hypersensitivity in mice

Relative to the Control group, the Model group showed significantly higher abdominal withdrawal reflex (AWR) scores at balloon distension volumes of 0.25, 0.35, and 0.50 mL (*P* < 0.05), reflecting a substantial rise in visceral sensitivity. Following drug intervention, all FRRTP treatment groups exhibited significantly reduced AWR scores at 0.25 mL, 0.35 mL, and 0.50 mL volumes compared to the Model group (*P* < 0.05). The RRTP group also showed significantly lower AWR scores at distension volumes of 0.35 and 0.50 mL (*P* < 0.05, Figure 3F). These results indicate that both FRRTP and RRTP alleviated visceral hypersensitivity.

#### 3.3.3 Effect of FRRTP on behavior in the forced swim test (FST)

**FIGURE 5:**
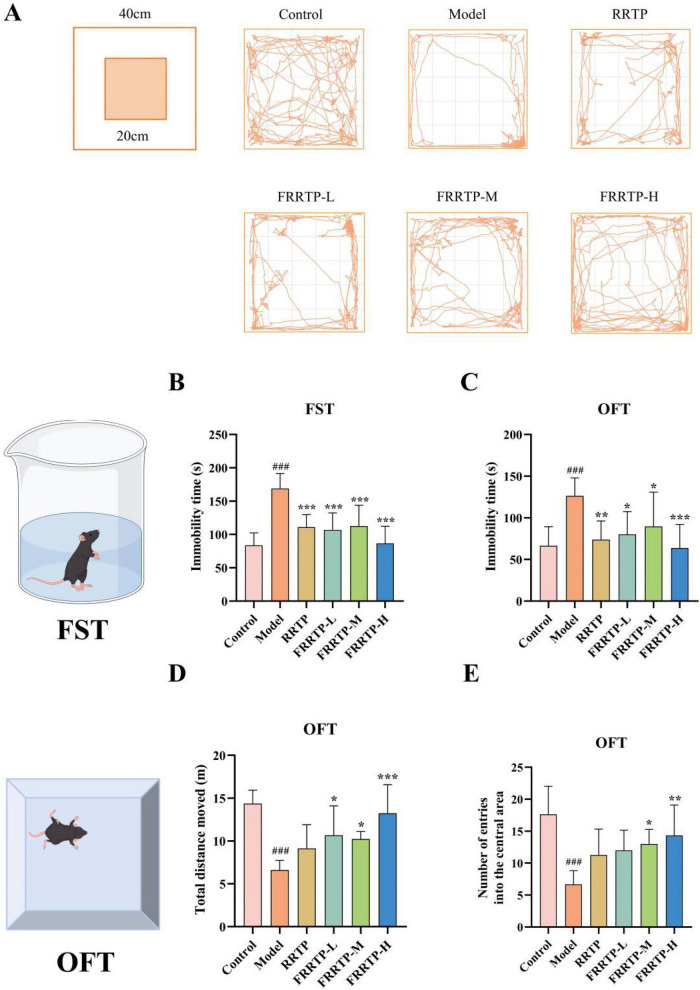
FRRTP alleviates anxiety-like behavior in IBS-D mice. **(A)** Representative movement trajectories of mice in the open field test. **(B)** Immobility time of mice in the forced swim test. **(C)** Immobility time of mice in the open field test. **(D)** Total distance moved by mice in the open field test. **(E)** Number of entries into the central area by mice in the open field test. (**^###^***P* < 0.001 vs. Control group; *P < 0.05, **P < 0.01, ***P < 0.001, vs. Model group).

The FST revealed significant differences in immobility duration among the groups (Figure 5B). Immobility time was significantly prolonged in the Model group compared with the Control group (*P* < 0.05). After FRRTP administration, a significant decrease in immobility duration was observed in the treatment groups compared with the Model group (*P* < 0.05). These data indicate that FRRTP reduced behavioral despair in the forced swim test, suggesting its antidepressant-like activity.

#### 3.3.4 Effects of FRRTP on behavior in the open field test (OFT)

Analysis of movement trajectories in the OFT revealed distinct behavioral patterns. Mice in the Control group initially explored the central zone and subsequently traversed most areas of the arena with smooth, continuous movement. In contrast, trajectories of mice in the Model group were largely confined to the periphery, exhibiting pronounced thigmotaxis (“wall-hugging”) and stereotypic cornering behavior. Representative trajectory maps are shown in Figure 5A.

Quantitative analysis revealed that, compared with the Control group, Model group mice spent significantly more time immobile and showed reduced total distance and fewer central area entries (*P* < 0.05). Treatment with RRTP or FRRTP significantly decreased immobility time compared with the Model group (*P* < 0.05, Figure 5C). Furthermore, all FRRTP doses significantly increased total distance moved (*P* < 0.05, Figure 5D), while FRRTP-M and FRRTP-H also significantly increased central area entries (*P* < 0.05, Figure 5E). These results indicate that FRRTP alleviated anxiety-like behaviors in IBS-D mice.

### 3.4 FRRTP ameliorates neurotransmitter dysregulation in IBS-D mice

**FIGURE 6:**
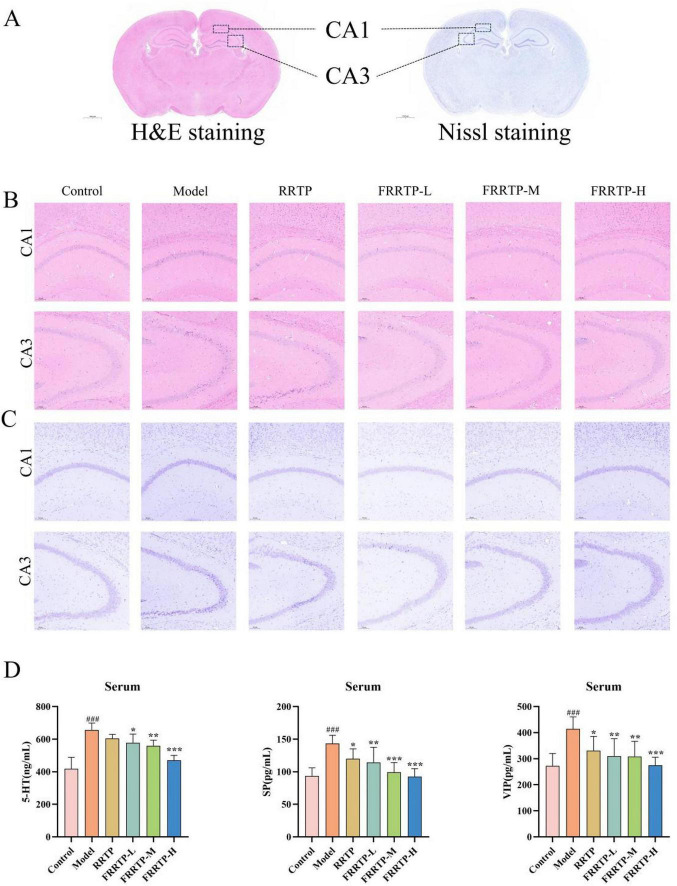
FRRTP alleviates hippocampal neuronal damage and peripheral neurotransmitter dysregulation in IBS-D mice. **(A)** Schematic diagram of the hippocampal subregions. **(B)** Representative H&E staining images of hippocampal tissue from each group (*n* = 3). Scale bar, 100 μm. **(C)** Representative Nissl staining images of hippocampal tissue from each group (*n* = 3). Scale bar, 100 μm. **(D)** Serum levels of 5-HT, SP and VIP. (**^###^***P* < 0.001 vs. Control group; *P < 0.05, **P < 0.01, ***P < 0.001, vs. Model group).

As shown in Figures 4E, 6D, the Model group exhibited significantly elevated levels of 5-HT, SP, and VIP in both serum and colon compared with the Control group (*P* < 0.05). In serum, all FRRTP doses (L, M, H) significantly reduced the levels of 5-HT, SP, and VIP relative to the Mod group (*P* < 0.05). RRTP treatment also significantly reduced serum SP levels (*P* < 0.05). In the colon, 5-HT levels were significantly reduced in the FRRTP-M and FRRTP-H groups (*P* < 0.05), and VIP levels were significantly reduced in the RRTP, FRRTP-L, FRRTP-M, and FRRTP-H groups (*P* < 0.05). Collectively, these findings indicate that FRRTP modulates the levels of 5-HT, SP, and VIP in both serum and colon, which may contribute to its therapeutic effects in IBS-D mice.

### 3.5 FRRTP alleviates colon and brain injury in IBS-D mice

Recognizing that IBS-D frequently correlates with pathological alterations in both brain and colon tissues, we investigated these organs in our experimental model. Relative to the Control group, the Model group exhibited a significant reduction in colon length. Treatment with FRRTP, especially at medium and high dosages (FRRTP-M, FRRTP-H), prevented this shortening compared with the Model group (*P* < 0.05; Figure 4B). In IBS-D mice, FRRTP also significantly curbed the rise in key pro-inflammatory cytokines (TNF-α, IL-1β, and IL-6) in colon tissue (*P* < 0.05, Figure 4D).

As revealed by H&E staining of colon tissue sections (Figure 4C), the Model group presented a significant loss of goblet cells along with disrupted mucosal architecture and mild inflammatory cell infiltration (lymphocytes and neutrophils) compared to the Control group. Notably, FRRTP administration significantly ameliorated the pathological changes induced by combined stress and *Senna Folium* extract administration. These findings indicate that FRRTP effectively mitigates colon injury in IBS-D mice.

As illustrated in Figure 6A (schematic diagram of the mouse hippocampal CA1 and CA3 regions), concurrently, H&E staining of the hippocampal region (Figure 6B) was used to assess cellular damage. Neurons in the Control group showed round, intact nuclei and orderly arrangement. In contrast, the Model group displayed shrunken neuronal soma, pyknotic and hyperchromatic nuclei, partial nuclear fragmentation, and other signs of cellular injury. FRRTP intervention at all doses resulted in largely preserved neuronal integrity. Nissl staining (Figure 6C) further demonstrated that, in contrast to the Control group, neurons in the Model group exhibited loosely arranged neurons with widened intercellular spaces, decreased cell numbers, abnormal morphology, nuclear shrinkage, and reduced Nissl substance. Compared to the Model group, all FRRTP treatment groups significantly alleviated these neuronal injuries.

### 3.6 Effects of FRRTP on the gut microbiota in IBS-D mice

#### 3.6.1 Alpha diversity analysis of gut microbiota

**FIGURE 7:**
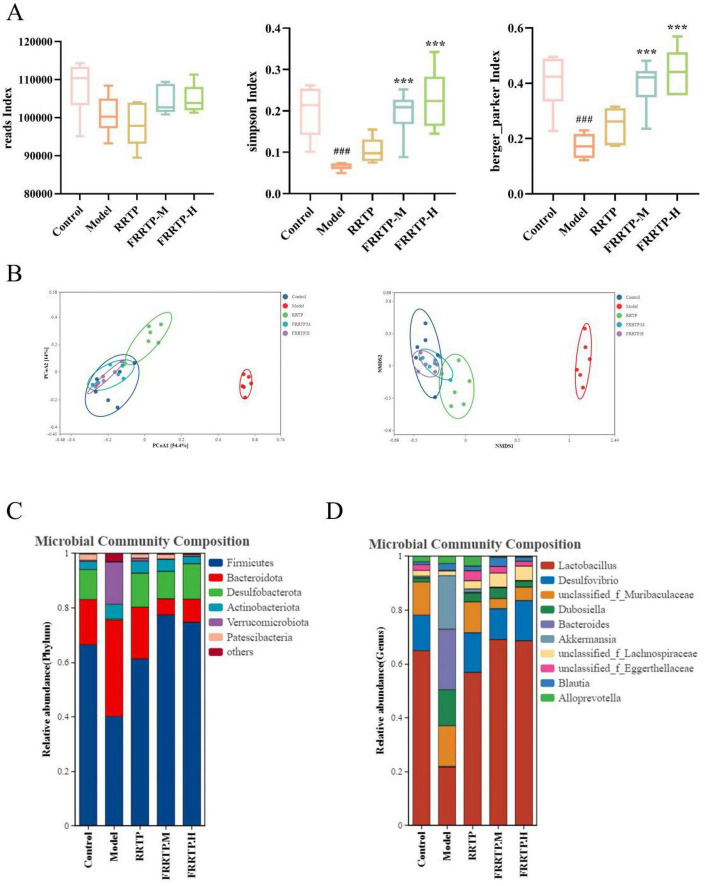
Effects of FRRTP on the gut microbiota in IBS-D mice (*n* = 6).**(A)** Analysis of *alpha* diversity indices (The index of reads, Simpson, berger_parker). **(B)** *Beta* diversity analysis visualized by Principal Coordinates Analysis (PCoA) and Non-metric Multidimensional Scaling (NMDS). **(C)** Relative abundance of the gut microbiota at the *phylum* level in each group. **(D)** Relative abundance of the gut microbiota at the *genus* level in each group. (###*P* < 0.001 vs Control group; ***P < 0.001, vs Model group).

Relative to the Control group, the Model group exhibited significant reductions in both the simpson and berger-parker indices of gut microbiota (*P* < 0.05). Although the difference in the reads index did not reach statistical significance, a decreasing trend was observed. Treatment with FRRTP-M and FRRTP-H partially reversed these reductions, as indicated by increased alpha diversity indices compared to the Model group. These results suggest that FRRTP may mitigate the loss of gut microbial diversity in IBS-D mice, although the effect was dose-dependent and not fully restored to Control levels (Figure 7A).

#### 3.6.2 Beta diversity analysis of gut microbiota

Beta diversity was assessed using PCoA and NMDS ordination methods based on unweighted UniFrac distances. Both approaches revealed partial separation among the experimental groups, suggesting potential differences in overall gut microbial community structure (Figure 7B). Samples from the FRRTP-M and FRRTP-H groups tended to cluster more tightly and showed a modest shift toward the Control group region; however, they did not fully converge with the Control cluster. These findings imply that FRRTP treatment may influence the global composition of the gut microbiota in IBS-D mice, but the effect appears to be moderate and dose-dependent, and the community structure was not fully normalized.

#### 3.6.3 Analysis of microbial community composition

At the phylum level (Figure 7C), the Model group showed a lower *Firmicutes/Bacteroidota* ratio than the Control group, driven by decreased *Firmicutes* and increased *Bacteroidota*. Each treatment regimen was associated with a shift toward the Control pattern; however, the magnitude of this shift varied, with FRRTP-M and FRRTP-H producing a more evident trend than RRTP. Even so, the phylum-level composition in treated groups did not fully match that of the Control group.

At the genus level (Figure 7D), compared with Controls, Model mice had less *Lactobacillus* but more *Bacteroides* and *Dubosiella.* All interventions increased Lactobacillus and reduced the other two genera to varying extents. Although the FRRTP-M and FRRTP-H groups showed greater changes than the RRTP group, individual mouse data revealed some overlap with the Model group’s profile, indicating that the restoration was not uniform across animals. Taken together, these observations suggest that FRRTP can partially reshape the gut microbiota of IBS-D mice, with medium and high doses showing more noticeable effects, yet the community structure was not completely normalized.

### 3.7 Effects of FRRTP on the metabolome in colon tissues and serum of IBS-D mice

**FIGURE 8:**
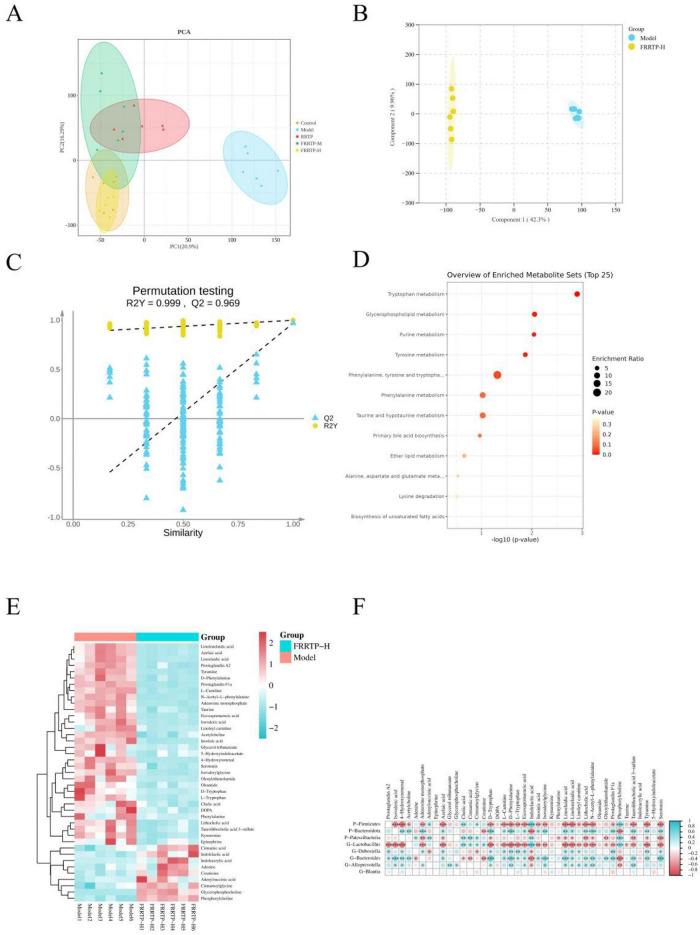
Effects of FRRTP on colonic metabolism in IBS-D mice. **(A)** PCA score plot of colon tissue metabolites. **(B)** OPLS-DA score plot comparing the Model and FRRTP-H groups. **(C)** Permutation test plot (*n* = 200) of the OPLS-DA model for the Model vs. FRRTP-H comparison. **(D)** KEGG pathway enrichment analysis of differential metabolites in the FRRTP-H group compared to the Model group. **(E)** Heatmap of differential metabolites between the Model and FRRTP-H groups. **(F)** Spearman correlation analysis between differential colon metabolites and gut microbiota.

**FIGURE 9:**
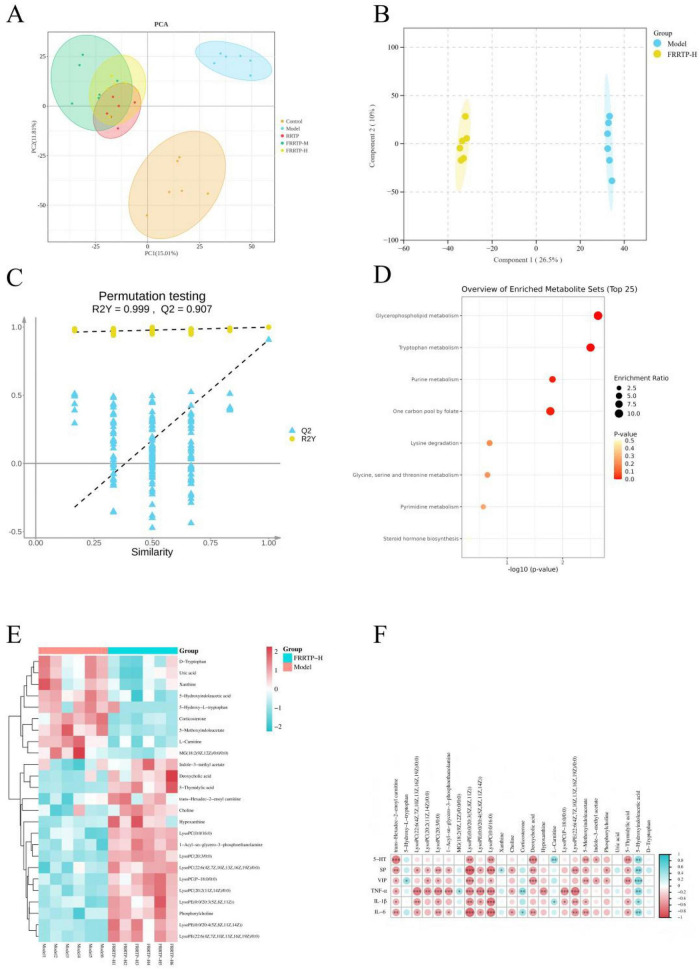
Effects of FRRTP on serum metabolism in IBS-D mice. **(A)** PCA score plot of serum metabolites. **(B)** OPLS-DA score plot comparing the Model and FRRTP-H groups. **(C)** Permutation test plot (*n* = 200) of the OPLS-DA model for the Model vs. FRRTP-H comparison. **(D)** KEGG pathway enrichment analysis of differential metabolites in the FRRTP-H group compared to the Model group. **(E)** Heatmap of differential metabolites between the Model and FRRTP-H groups. **(F)** Spearman correlation analysis between differential serum metabolites and inflammatory cytokines/neurotransmitters.

To investigate the alterations in endogenous metabolites following FRRTP intervention, we conducted an untargeted metabolomic analysis using serum and colon samples collected from each group (see Figures 8, 9). Significant metabolic disturbance in IBS-D mice was evident from the PCA results, which displayed a discernible separation between groups on the second principal component (PC2) for both colon tissue and serum (Figures 8A, 9A). Following FRRTP intervention, the metabolic profiles of treated groups converged towards the Control and away from the Model group, with the most evident reversal observed in the FRRTP-H group. OPLS-DA was subsequently performed between the Model and FRRTP-H groups (Figures 8B,C, 9B,hyperref[F9]C), confirming significant metabolic separation. A permutation test (200 iterations) validated the robustness and reliability of the OPLS-DA models without overfitting.

Differential metabolites were screened using *t*-tests (*P* < 0.05) with thresholds of variable importance in projection (VIP) > 1.0 and fold change (FC) < 0.8 or > 1.2. This identified 41 and 25 significant metabolites in colon tissue and serum, respectively. Validation against the HMDB database and visualization by heatmap showed that in colon tissue, 9 metabolites were upregulated and 32 were downregulated (Figure 8E), while in serum, 16 were upregulated and 9 were downregulated (Figure 9E). Pathway enrichment analysis using MetaboAnalyst revealed 12 and 7 significantly impacted pathways in colon and serum, respectively. Shared pathways included tryptophan, glycerophospholipid, and purine metabolism. In colon tissue, several metabolic pathways were significantly altered, including tryptophan metabolism; phenylalanine, tyrosine, and tryptophan biosynthesis; taurine and hypotaurine metabolism; and purine metabolism (Figure 8D). In serum, tryptophan metabolism, purine metabolism, and glycerophospholipid metabolism exhibited significant alterations (Figure 9D).

To integrate these findings, Spearman correlation analyses were conducted. Correlations were observed between gut microbiota at the genus/phylum level and differential metabolites in the colon (Figure 8F), while neurotransmitters and inflammatory factors were correlated with serum differential metabolites (Figure 9F). Key findings included: *Firmicutes* and *Lactobacillus* were negatively correlated with 23 colon metabolites (e.g., serotonin, L-tryptophan) and positively correlated with 6 others (e.g., phosphorylcholine); *Bacteroides* showed the opposite correlation pattern. In serum, neurotransmitters and inflammatory factors positively correlated with metabolites such as 5-HTP and corticosterone, and negatively correlated with various lysophospholipids and choline compounds.

Collectively, these results demonstrate that FRRTP ameliorates systemic metabolic dysregulation in IBS-D mice and reveal multi-level crosstalk among the gut microbiota, host metabolism, and neuro-immune signals.

### 3.8 The remodeling effect of FRRTP treatment on abnormal brain metabolism in IBS-D mice

**FIGURE 10:**
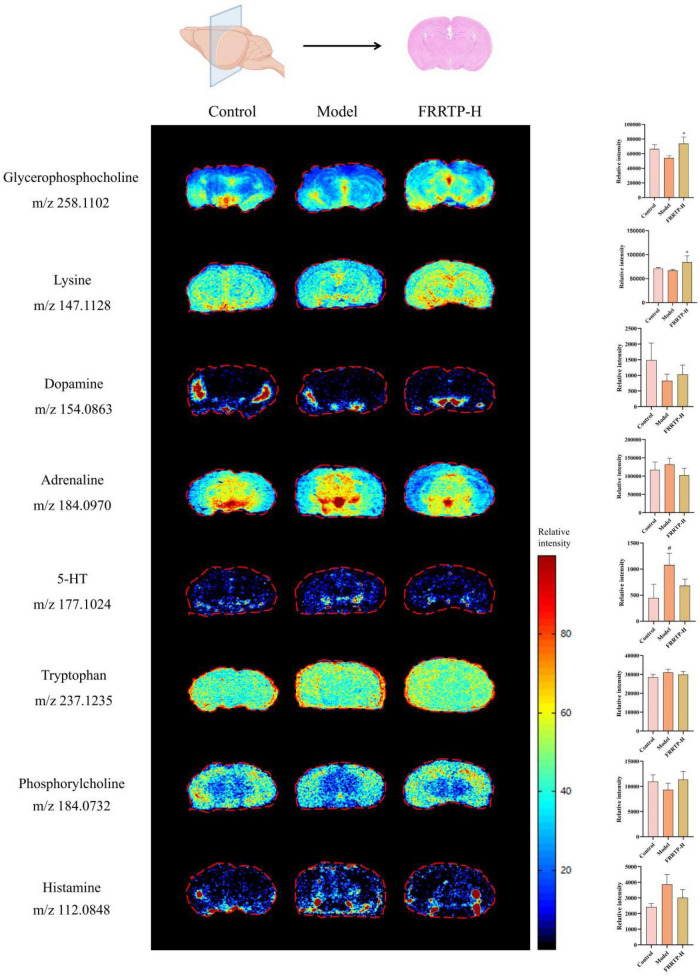
Effects of FRRTP on endogenous brain metabolites in IBS-D mice. (#*P* < 0.05 vs Control group; *P < 0.05, vs Model group).

Spatial metabolomic profiling using mass spectrometry imaging (MSI) was performed to map the distribution of specific compounds in mouse brains. Following data acquisition and background subtraction, ion images for compounds of interest were reconstructed by extracting specific m/z values corresponding to their protonated ions in positive mode. This approach generated spatial distribution maps for glycerophosphocholine, lysine, dopamine, adrenaline (epinephrine), serotonin (5-HT), tryptophan, phosphorylcholine, and histamine. Compared with the Control group, the Model group showed reduced concentrations of glycerophosphocholine, lysine, dopamine, and phosphorylcholine, and elevated concentrations of adrenaline, serotonin, tryptophan, and histamine. These alterations suggest dysregulation of monoaminergic signaling, activation of the stress response system, imbalance in cholinergic metabolism, and abnormalities in amino acid metabolism– processes implicated in visceral sensation, mood regulation, and cognition. Notably, FRRTP intervention restored the levels and spatial distribution patterns of these metabolites toward those of the Control group. This reversal of the disordered metabolic landscape indicates that FRRTP ameliorates IBS-D, at least in part, by rectifying brain metabolism associated with its pathology (Figure 10).

## 4 Discussion

Irritable bowel syndrome (IBS) is a functional gastrointestinal disorder characterized by chronic abdominal pain or discomfort, resulting from dysregulated gut-brain communication involving multiple contributing factors. The diarrhea-predominant subtype (IBS-D) is the most prevalent variant in China. IBS-D is a non-organic gastrointestinal disorder, with primary symptoms including abdominal pain, bloating, or discomfort accompanied by altered bowel habits. Patients often experience concurrent psychiatric disorders such as anxiety. The exact etiology and pathogenesis of IBS-D remain incompletely understood, but current research suggests that visceral hypersensitivity, psychosocial stress, gut-brain axis dysfunction, low-grade inflammation, gut microbiota dysbiosis, and genetic factors may be involved. Among these, dysregulation of the nervous system mediated by the gut-brain axis, leading to visceral hypersensitivity and smooth muscle hyperreactivity, is considered the primary mechanism (29).

Establishing an animal model that closely mimics clinical symptoms is crucial for in-depth investigation of IBS-D. Existing modeling methods include chronic unpredictable mild stress, chronic restraint stress combined with tail-pinching stress, intragastric administration of *Senna Folium* aqueous extract combined with restraint stress, and acetic acid enema combined with maternal separation (30). Currently, multifactorial combined modeling is the mainstream approach because it better simulates the complex clinical manifestations of IBS-D, such as diarrhea and abdominal pain (31). In this study, we successfully established an IBS-D mouse model using *Senna Folium* aqueous extract combined with restraint stress and tail-pinching stress. This model is operationally simple, stable, and highly reproducible. During model induction, Senna Folium aqueous extract induces colonic dysfunction (32), while restraint stress and tail-pinching stress evoke visceral hypersensitivity and mood disorders in mice (33, 34), consistent with previous reports. Therefore, this model effectively mimics the core clinical symptoms of IBS-D, namely abdominal pain and diarrhea.

*Rosa roxburghii* Tratt, recognized for its dual use as both food and medicine, has a documented history in traditional medicine for regulating digestive function. It modulates gut microecology and exerts antioxidative and anti-inflammatory effects, indicating its therapeutic potential for IBS-D. However, traditional processing methods such as juicing or aqueous extraction are inefficient at fully liberating its bioactive constituents, thereby limiting bioavailability and overall efficacy. Probiotic fermentation has emerged as a transformative strategy to address this limitation. Leveraging microbial biotransformation—a well-established principle for enhancing the bioactivity and reducing potential toxicity of herbal materials—we applied co-fermentation using *Saccharomyces cerevisiae* and *Bacillus subtilis* to *Rosa roxburghii* Tratt pulp. This approach is supported by studies showing that fermentation can significantly alter the profile and potency of bioactive compounds, notably enhancing *in vitro* antioxidant activity (35). Therefore, by investigating the chemical changes induced by fermentation and the subsequent protective effects against IBS-D, this study aims to develop a safe, reliable, food-derived intervention for alleviating IBS-D symptoms.

This research represents the first systematic effort to elucidate the comprehensive beneficial effects of both unfermented (RRTP) and fermented (FRRTP) pulp extracts of *Rosa roxburghii* Tratt on mice with IBS-D, while also exploring the underlying mechanisms. Our findings showed that fermentation significantly increased the concentration of bioactive compounds in RRTP. Compared to RRTP, FRRTP was more effective in alleviating diarrhea, abdominal discomfort, and mood disturbances in IBS-D mice, indicating that co-fermentation enhances the bioactivity of RRTP. Compositional analysis confirmed the biotransformation induced by fermentation, which likely constitutes the material basis for the superior efficacy of FRRTP. In our study, FRRTP treatment in IBS-D mice effectively mitigated reduced weight gain, decreased fecal water content and Bristol stool scores, alleviated anxiety-like behaviors, reduced visceral hypersensitivity, and ameliorated colon shortening and thymus atrophy.

H&E staining of colon tissue showed that FRRTP significantly alleviated colonic injury in IBS-D mice by reducing inflammatory cell infiltration and apoptosis. The pathogenesis of IBS-D involves intestinal epithelial barrier impairment, immune cell activation, and the release of inflammatory cytokines. Specifically, pro-inflammatory cytokines such as IL-1β, IL-6, and TNF-α are elevated, contributing to low-grade intestinal inflammation (36, 37). FRRTP administration reduced the levels of these pro-inflammatory cytokines in the colon, demonstrating its efficacy in reducing inflammation and alleviating IBS-D.

The pathophysiology of IBS-D is also closely associated with gut microbiota dysbiosis, primarily manifested as reduced abundance of beneficial bacteria, overgrowth of pathogenic bacteria, and decreased microbial diversity (38). Gut microbiota dysbiosis can disrupt the colonic mucosal barrier, increase intestinal permeability, and trigger immune dysregulation, thereby sensitizing neuroendocrine and stress-related behaviors (39). In this study, FRRTP intervention induced a significant positive remodeling of the gut microbiota structure in IBS-D mice, with the *Bacteroidota/Firmicutes* ratio tending to normalize. Notably, the abundance of *Lactobacillus* was significantly increased. *Lactobacillus* has been a focus of research on intestinal diseases and is commonly used in their treatment, as it can regulate intestinal pH, inhibit adhesion of harmful bacteria, and enhance the integrity of the intestinal mucosal barrier. Meanwhile, the abundances of opportunistic pathogens such as *Bacteroides* and *Dubosiella* were markedly reduced, decreasing inflammatory stimulation of the intestinal mucosa and the risk of intestinal motility disorders. These results suggest that FRRTP may improve gut microbiota imbalance in IBS-D mice by directly inhibiting pathogen growth or providing a favorable environment for beneficial bacteria, consistent with previous strategies for modulating gut microbiota, such as probiotic and dietary interventions. The relative abundance of *Lactobacillus* may be a therapeutic target for IBS-D.

The bidirectional communication between the gut microbiota and the central nervous system—referred to as the “microbiota–gut–brain axis”—is now recognized as a core mechanism underlying the development and progression of neurobehavioral comorbidities in IBS-D. As described above, FRRTP intervention increased the abundance of beneficial bacteria (e.g., *Lactobacillus*) and reduced the proportion of opportunistic pathogens (e.g., *Bacteroides, Dubosiella*), while concurrently improving anxiety-like behaviors, alleviating hippocampal neuronal damage, and restoring aberrant distribution of key cerebral metabolites. These highly correlated observations suggest that FRRTP may exert coordinated regulation via the microbiota–gut–brain axis. The influence of gut microbiota on central nervous system function involves three main pathways. Regarding the metabolite pathway, gut microbiota can convert tryptophan into signaling molecules such as indole derivatives, thereby modulating 5-HT synthesis. Dysbiosis shifts tryptophan metabolism toward the kynurenine pathway and reduces central 5-HT synthesis (40, 41). In this study, FRRTP significantly increased the abundance of *Lactobacillus*, which is known to convert tryptophan into aryl hydrocarbon receptor ligands (e.g., indole-3-aldehyde) and thereby regulate gut immunity and local 5-HT synthesis (42, 43). Regarding the immune-inflammatory pathway, dysbiosis can trigger gut-derived inflammation. IBS-D is characterized by low-grade intestinal inflammation and systemic immune activation. Pro-inflammatory cytokines can cross the blood–brain barrier, activate microglia, and induce neuroinflammation and anxiety/depressive-like behaviors (44). In our study, FRRTP intervention significantly reduced colonic inflammatory cytokine levels and concurrently alleviated hippocampal neuronal damage, indicating that FRRTP may indirectly protect central neurons by suppressing gut-derived inflammation. Regarding the vagal pathway, vagal nerve endings sense microbial metabolites, 5-HT, and other signals, transmitting them to the nucleus tractus solitarius and upstream limbic system. Patients with IBS often exhibit reduced vagal tone (45, 46).

Taken together, these findings suggest that FRRTP exerts its therapeutic effects through microbiota remodeling across multiple pathways, providing an initial chain of evidence for its action via the microbiota–gut–brain axis in treating IBS-D. To further validate the above metabolite pathway, we next analyzed the impact of microbiota changes on host metabolic status.

Shifts in the gut microbiome can further modulate the metabolic status of the host. Preliminary results showed that FRRTP exerted a dose-dependent therapeutic effect on IBS-D, with the high-dose group (FRRTP-H) being the most effective; therefore, this dose was selected for subsequent analyses. Metabolomics of colon and serum revealed that FRRTP significantly regulated the tryptophan, glycerophospholipid, and purine metabolic pathways. Tryptophan metabolites, such as 5-HT, are key messengers in the gut-brain axis, and their dysregulation is associated with visceral hypersensitivity and mood disorders in IBS-D. In IBS-D model mice, 5-HT levels were elevated in both serum and colon tissues, indicating intestinal accumulation of 5-HT, which is consistent with the clinical features of accelerated intestinal motility and heightened visceral pain sensitivity in IBS-D patients (47). FRRTP intervention reduced 5-HT concentrations in both colon and serum, suggesting that FRRTP ameliorates neurotransmitter imbalance by regulating tryptophan metabolism. Glycerophospholipids are core components of the intestinal epithelial cell membrane and precursors of signaling molecules; their metabolic imbalance is a key contributor to intestinal structural and functional damage in IBS-D. Our colon and serum metabolomic analyses showed that FRRTP repaired this abnormal glycerophospholipid metabolism, thereby alleviating IBS-D. Furthermore, studies have shown that in stress-induced IBS-D, stress leads to excessive xanthine production, which in turn alters the gut microbiota and promotes spermidine overproduction, ultimately causing colonic smooth muscle hypercontraction and increased defecation frequency. Our study found that FRRTP ameliorated purine metabolic disorders in IBS-D mice, reducing levels of metabolites such as xanthine and hypoxanthine. This restorative effect may be mediated through two mechanisms: on the one hand, via its anti-inflammatory and immunomodulatory actions, FRRTP may reduce stress-induced aberrant T-cell activation, thereby decreasing xanthine production at its source; on the other hand, by modulating the gut microbiota, FRRTP may disrupt the conversion pathway from xanthine to excess spermidine, ultimately restoring normal intestinal motility.

Current research indicates that the long-term low-grade inflammation in IBS-D can sensitize local nerve fibers and further lead to central sensitization; therefore, IBS-D is considered a gut-brain axis disorder (37). As a high-speed bidirectional pathway connecting the gut and the brain, the vagus nerve plays a crucial role in signal transmission and regulation in IBS-D and may be a key mediator of the effects of the fermented product in this study. Vagus nerve dysfunction, particularly reduced vagal tone, has been confirmed to be closely associated with the pathogenesis of functional gastrointestinal disorders such as IBS (48). In the IBS-D state, the vagus nerve continuously receives abnormal signals from the disturbed intestinal microenvironment, which in turn affects brain regions and may exacerbate visceral pain perception and negative emotional responses (49), conversely, central states such as stress and mood can also influence the intestinal environment via the vagus nerve, forming a vicious cycle.

Studies have shown that mood disorders and visceral hypersensitivity in IBS-D are associated with abnormal expression of the brain-gut peptides 5-HT, SP, and VIP, which are key components of the gut-brain axis (50, 51). In IBS-D, elevated levels of 5-HT, SP, and VIP promote intestinal motility, affect secretory function, and increase sensitivity to painful stimuli. In this study, FRRTP intervention reduced the levels of 5-HT, SP, and VIP in both the colon and serum of mice, indicating that FRRTP improved the secretion of these brain-gut peptides, thereby alleviating visceral hypersensitivity and emotional disturbances in IBS-D mice, with effects superior to those of the RRTP group. Meanwhile, H&E and Nissl staining of brain tissue showed that FRRTP treatment significantly ameliorated hippocampal neuronal damage and apoptosis in IBS-D mice. These findings confirm that FRRTP possesses anxiolytic and mood-improving effects.

To further investigate the effect of FRRTP on brain metabolism and provide evidence for the metabolic coordination along the gut-brain axis, this study employed spatial metabolomics (AFADESI-MSI) to visualize IBS-D-related endogenous metabolites in the mouse brain. Our data showed that FRRTP regulated the metabolism of monoamine neurotransmitters and their precursors. The central 5-HT system, which is central to the regulation of mood, pain perception, and gastrointestinal motility, was partially improved after FRRTP intervention, although the absolute content of 5-HT changed little and its cerebral distribution disorder was partially corrected. This finding echoes a previous study (52), which reported that 5-HT levels were elevated in the gut, brain, and serum of IBS-D mice, and that reducing 5-HT levels alleviated IBS-D symptoms. Dopamine regulates emotional responses via reward pathways, and its imbalance can lead to negative emotions such as anxiety in IBS-D mice (53). Adrenaline and histamine are associated with stress responses, visceral hypersensitivity, and intestinal motility dysfunction (54). Our data indicated that FRRTP corrected the metabolic disturbances of these monoamine neurotransmitters and their precursors in the brain, directly addressing the core pathology of IBS-D—affective, sensory, and motor dysregulation.

Tryptophan is the sole precursor of 5-HT synthesis, and its cerebral availability is the key limiting factor for central 5-HT production (55). Lysine, as an essential amino acid, is not only a building block for protein synthesis but also participates in energy metabolism via its metabolites; lysine deficiency impairs learning and memory and can induce agitation and other abnormal behaviors (56). Our study showed that FRRTP improved amino acid metabolism and regulated neurotransmitter synthesis, thereby providing structural support for central regulation of gut function. Imbalanced glycerophospholipid metabolism can compromise the stability of brain cell membranes and may be closely associated with anxiety-like behaviors and visceral hypersensitivity in IBS-D mice. Studies have shown that hippocampal glycerophospholipid metabolites are markedly abnormal in depressed mice, indicating a significant correlation between glycerophospholipid changes and anxiety/depression-like phenotypes (57, 58). Mass spectrometry imaging further revealed significantly abnormal levels of the key glycerophospholipid intermediates glycerophosphocholine and phosphorylcholine in the brains of IBS-D model mice, and FRRTP intervention effectively rectified this disorder, providing core evidence for the metabolic synergy along the gut-brain axis.

The multi-omics data presented above indicate that the beneficial effects of FRRTP on IBS-D mice operate through a three-step synergistic mechanism: fermentation-driven pre-transformation, gut microbiota reactivation, and systemic regulation via absorption of metabolites into the bloodstream. First, the flavonoids and polyphenols in FRRTP exist mainly in conjugated forms with limited bioavailability, and the gut microbiota play a crucial role in polyphenol release (59). In this study, co-fermentation with *Saccharomyces cerevisiae* and *Bacillus subtilis* was employed to achieve pre-activation of the bioactive components. Ran et al. demonstrated that microbial fermentation can convert macromolecular polyphenols into small-molecular phenolic acids (e.g., ferulic acid, p-hydroxybenzoic acid) through deglycosylation (60), while fermentation enzymes improve the solubility and stability of these small-molecular products, thereby facilitating absorption (61). This fermentation pre-treatment shifts part of the colonic transformation to an earlier stage, reducing the metabolic burden on the host gut microbiota and simultaneously increasing the initial bioavailability of the active components. Second, the incompletely transformed conjugated polyphenols enter the colon and undergo a second-phase transformation. Zhou et al. reported that solid-state fermented *Rosa roxburghii* Tratt pomace modulates the gut microbiota structure in rats, decreasing the *Firmicutes/Bacteroidetes* ratio and enriching butyrate-producing bacteria such as *Ruminococcus, Allobaculum*, and *Bifidobacterium* (62), butyrate and other short-chain fatty acids are known to enhance intestinal barrier function. Consistently, our 16S rRNA sequencing results showed an increase in beneficial bacteria such as *Lactobacillus* and a decrease in opportunistic pathogens including *Bacteroides* and *Dubosiella*. The review by Mahdi et al. further pointed out that polyphenols not absorbed in the small intestine need to be converted by the gut microbiota into simpler phenolic products to increase their bioavailability and enter the systemic circulation (63). Our metabolomics data confirmed alterations in the tryptophan, purine, and glycerophospholipid pathways, establishing the central role of the gut microbiota in FRRTP transformation. Finally, the small-molecule metabolites generated by microbial transformation are absorbed into the blood and distributed throughout the body. Zhou et al.’s serum and fecal metabolomics demonstrated that fermented *Rosa roxburghii* Tratt pomace induces changes in flavonoid-related metabolites and tryptophan metabolites (e.g., serotonin, indole-3-lactic acid) (62), our spatial metabolomics further revealed that FRRTP ameliorates the disturbed cerebral distribution of key metabolites such as 5-HT and tryptophan, directly participating in the regulation of mood and visceral sensation. These findings suggest that microbiota-mediated metabolic transformation effects can be transmitted to the central nervous system via the gut-brain axis.

**FIGURE 11:**
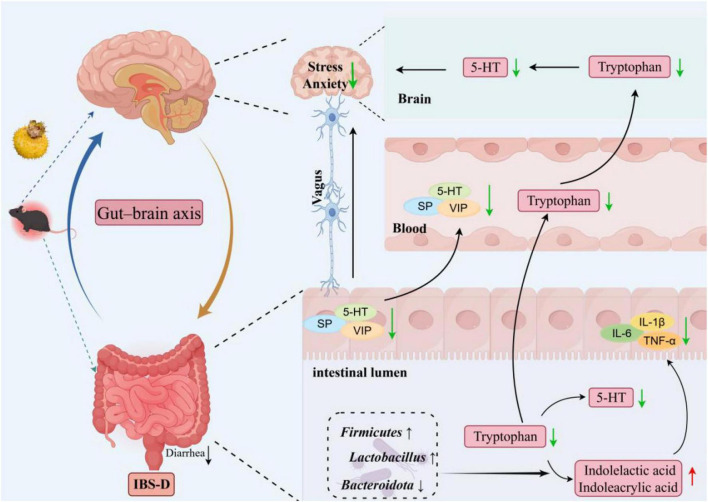
Schematic illustration of the underlying mechanism by which FRRTP alleviates IBS-D in mice via the gut-brain axis (by Figdraw.com).

In summary, FRRTP acts directly on the gut and indirectly regulates host metabolism through microbiota remodeling, synergistically protecting the intestinal barrier, reducing inflammation, and restoring gut-brain axis function, thereby effectively alleviating IBS-D (Figure 11).

## 5 Conclusion

This study reveals that co-fermentation of *Rosa roxburghii* Tratt pulp with *Saccharomyces cerevisiae* and *Bacillus subtilis* represents a promising strategy for remodeling its bioactive profile. The resulting ethanolic extract (FRRTP) demonstrated significant amelioration of diarrhea-predominant irritable bowel syndrome (IBS-D) in a mouse model. Mechanistically, the beneficial effects appear to be mediated through a sequential axis involving gut microbiota remodeling, modulation of microbial and host metabolism, and subsequent restoration of intestinal barrier function, inflammatory balance, and gut-brain axis signaling—an ecosystem-level cascade that aligns with emerging microbiome-centered therapeutic paradigms.

Our findings highlight the potential application of fermented *Rosa roxburghii* Tratt pulp as a functional food ingredient or a prebiotic-like supplement for managing IBS-D and other microbiota-related gastrointestinal disorders. The relatively low cost and wide availability of this fruit in southwest China further support its development into an accessible dietary intervention, particularly for populations seeking non-pharmacological options.

Beyond the specific extract, the co-fermentation approach itself may offer a generalizable platform for valorizing underutilized fruits or agricultural by-products. Conceptually, this work supports a shift from reductionist single-compound thinking toward holistic, microbiota-targeted strategies. Future research should focus on isolating the key active constituents, establishing causal relationships—for instance, through fecal microbiota transplantation (FMT) experiments to directly test whether FRRTP-shaped gut microbiota recapitulates the therapeutic effects—as this was not performed in the present study. Additionally, germ-free or genetic models and chronic stress-induced IBS-D models will help address current limitations regarding mechanistic causality and model complexity, thereby paving the way for clinical translation.

## Data Availability

All data presented in the study have been deposited in the NCBI database under BioProject accession number PRJNA1469534: https://www.ncbi.nlm.nih.gov/bioproject/PRJNA1469534.
