# Supplementary material for: Co-fermentation of Rosa roxburghii Tratt pulp by Saccharomyces cerevisiae and Bacillus subtilis alleviates diarrhea-predominant irritable bowel syndrome in mice by reshaping gut microbiota and host metabolism
Source: Front Nutr. 2026 Jun 5;13:1854409. doi: 10.3389/fnut.2026.1854409 (PMC13279404; doi:10.3389/fnut.2026.1854409)
Supplement: Supplementary file 1 [file Supplementary_file_1.docx]

Supplementary Material S1

S1.Methods for the Determination of Chemical Constituents

S1.1 Determination of Total Polyphenol Content

The total polyphenol content was determined using the Folin-Ciocalteu method with appropriate modifications. Four extracts were accurately weighed to prepare test solutions at specified concentrations. An aliquot of each solution was transferred to a 10 mL brown volumetric flask, followed by the addition of 2.5 mL of a 10% Folin-Ciocalteu reagent. After thorough mixing and a reaction period of 5 minutes, 2 mL of a 10% Na₂CO₃ solution was added. The mixture was then diluted to the mark with ultrapure water, mixed thoroughly, and allowed to react for 60 minutes at room temperature in the dark. Finally, the absorbance was measured at 760 nm using a UV-visible spectrophotometer. Gallic acid was used as the standard for quantification.

S1.2 Determination of Total Flavonoid Content

The total flavonoid content was determined using the aluminum nitrate-sodium nitrite colorimetric method with slight modifications. Four extracts were accurately weighed to prepare test solutions at specified concentrations. An aliquot of each test solution was transferred to a 25 mL volumetric flask, followed by the addition of 1 mL of a 5% sodium nitrite solution. After thorough mixing, the mixture was allowed to react for 6 minutes. Subsequently, 1 mL of a 10% aluminum nitrate solution was added, the mixture was mixed again, and allowed to stand for another 6 minutes. Finally, 5 mL of a 10% sodium hydroxide solution was added, and after mixing, the solution was diluted to the mark with ultrapure water. The colored solution was allowed to develop for 30 minutes before its absorbance was measured at 510 nm using a UV-visible spectrophotometer. Rutin was used as the standard for quantification.

S1.3 Determination of Total Triterpene Content

The total triterpenoid content was determined using the vanillin-glacial acetic acid method. Four extracts were accurately weighed to prepare test solutions at specified concentrations. An aliquot of each test solution was transferred to a stoppered test tube and the solvent was evaporated to dryness in a water bath. Subsequently, 0.4 mL of a 5% vanillin-glacial acetic acid solution and 1 mL of perchloric acid were added. The mixture was heated at 60℃ in a water bath for 15 minutes. After the reaction was complete, the tube was cooled in an ice-water bath. Then, 5 mL of glacial acetic acid was added, the solution was mixed thoroughly and allowed to stand for 10 minutes. Finally, the absorbance was measured at 543 nm using a UV-visible spectrophotometer. Ursolic acid was used as the standard for quantification.

S1.4 Total Polysaccharide Content Determination

The total polysaccharide content was determined using the phenol-sulfuric acid method with minor modifications. Briefly, four extracts were accurately weighed and dissolved to prepare test solutions at specified concentrations. An aliquot of each test solution was pipetted into a stoppered test tube and diluted to 2 mL with ultrapure water. Then, 1 mL of a 5% phenol solution was added and mixed thoroughly, followed immediately by the addition of 5 mL of concentrated sulfuric acid. The mixture was vortexed vigorously to ensure homogeneity. It was then allowed to react at room temperature in the dark for 10 minutes, placed in a boiling water bath for 20 minutes, and rapidly cooled to room temperature in an ice bath. The absorbance of the solution was measured at 490 nm using a UV-visible spectrophotometer. Glucose was used as the standard for quantification.

S1.5 Determination of Ellagic Acid Content

The quantification of ellagic acid (both free and total) was performed using an Ultimate 3000 high-performance liquid chromatography (HPLC) system.Sample Preparation: Four extracts were accurately weighed to prepare test solutions at a specified concentration. The solution was centrifuged at 10,000 rpm for 10 minutes. The supernatant was filtered through a 0.22 μm microporous membrane to obtain the free ellagic acid sample. For the determination of total ellagic acid, an aliquot (10 mL) of the test solution was mixed with 10 mL of acidified methanol (containing 1.2 mol/L hydrochloric acid). The mixture was weighed, refluxed at 85°C for 6 hours, cooled to room temperature, and then reweighed. The weight loss was compensated by adding methanol. The resulting solution was diluted 10-fold with methanol and filtered through a 0.22 μm membrane prior to HPLC analysis.

Chromatographic Conditions: Separation was achieved on a Symmetry C18 column (4.6 mm × 250 mm, 5 µm) maintained at 35°C. The mobile phase consisted of (A) acetonitrile and (B) 0.25% aqueous formic acid, delivered at a flow rate of 0.8 mL/min. The injection volume was 10 µL, and detection was performed at 254 nm. The following gradient program was used: 0–2 min, 6% A (94% B); 2–3 min, 6–16% A (94–84% B); 3–10 min, 16–20% A (84–80% B); 10–20 min, 20–6% A (80–94% B).

S1.6 Determination of Catechin Content

The catechin content was determined by high-performance liquid chromatography (HPLC).Sample Preparation: Test solutions were prepared at a specified concentration from four accurately weighed extracts. Each solution was centrifuged at 10,000 rpm for 10 minutes, and the supernatant was filtered through a 0.22 μm microporous membrane prior to HPLC analysis.

Chromatographic Conditions: Analysis was performed on a Symmetry C18 column (4.6 mm × 250 mm, 5 µm) maintained at 35°C. The mobile phase consisted of acetonitrile (A) and 0.3% aqueous formic acid (B), delivered at a flow rate of 1.0 mL/min. The injection volume was 20 µL, and detection was carried out at 280 nm. The following gradient elution program was used: 0–5 min, 5–10% A (95–90% B); 5–7 min, 10–15% A (90–85% B); 7–9 min, 15–20% A (85–80% B); 9–18 min, 15–5% A (85–95% B).

S1.7 Determination of Luteolin Content

The luteolin content was determined by high-performance liquid chromatography (HPLC).Sample Preparation: A test solution was prepared at a specific concentration from four accurately weighed extracts. The solution was centrifuged at 10,000 rpm for 10 minutes, and the supernatant was filtered through a 0.22 μm microporous membrane prior to HPLC injection.

Chromatographic Conditions: Separation was achieved using a Symmetry C18 column (4.6 mm × 250 mm, 5 µm) maintained at 30°C. The isocratic mobile phase consisted of acetonitrile and a 0.1% phosphoric acid aqueous solution (40:60, v/v), delivered at a flow rate of 1.0 mL/min. The injection volume was 20 µL, and detection was performed at 350 nm.

S1.8 Determination of Kaempferol Content

The Kaempferol content was determined by high-performance liquid chromatography (HPLC). Sample Preparation: A test solution was prepared at a specific concentration from four accurately weighed extracts. The solution was centrifuged at 10,000 rpm for 10 minutes, and the resulting supernatant was filtered through a 0.22 μm microporous membrane prior to HPLC injection.

Chromatographic Conditions: The analysis was performed using a Symmetry C18 column (4.6 mm × 250 mm, 5 µm) maintained at 30°C. An isocratic mobile phase of acetonitrile and 0.2% phosphoric acid aqueous solution (35:65, v/v) was delivered at a flow rate of 1.0 mL/min. The injection volume was 10 µL, and detection was carried out at a wavelength of 350 nm.

S1.9 Determination of Rutin Content

The Rutin content was determined by high-performance liquid chromatography (HPLC). Sample Preparation: Test solutions were prepared at specified concentrations from four accurately weighed extracts. Each solution was centrifuged at 10,000 rpm for 10 minutes, and the supernatant was filtered through a 0.22 μm microporous membrane prior to HPLC injection.

Chromatographic Conditions: The analysis was performed on a Symmetry C18 column (4.6 mm × 250 mm, 5 µm) maintained at 30°C. The mobile phase consisted of methanol (A) and a 0.2% phosphoric acid aqueous solution (B), delivered at a flow rate of 1.0 mL/min. The injection volume was 20 µL, and detection was carried out at 360 nm. The following gradient elution program was used: 0–5 min, 40–60% A (60–40% B); 5–10 min, 60–90% A (40–10% B); 10–12 min, 90–40% A (10–60% B)

S1.10 Determination of Quercetin Content

The Quercetin content was determined by high-performance liquid chromatography (HPLC).Sample Preparation: Test solutions were prepared at specified concentrations from four accurately weighed extracts. Each solution was centrifuged at 10,000 rpm for 10 minutes, and the supernatant was filtered through a 0.22 μm microporous membrane prior to HPLC injection.

Chromatographic Conditions: The analysis was performed on a Symmetry C18 column (4.6 mm × 250 mm, 5 µm) maintained at 35°C. The mobile phase consisted of methanol (A) and a 0.2% phosphoric acid aqueous solution (B), delivered at a flow rate of 1.0 mL/min. The injection volume was 20 µL, and detection was carried out at 360 nm. The following gradient elution program was applied: 0–5 min, 50–70% A (50–30% B); 5–10 min, 70–90% A (30–10% B); 10–15 min, 90–100% A (10–0% B); 15–16 min, 100–50% A (0–50% B).

S1.11 Determination of Gallic Acid Content

The Gallic Acid content was determined by high-performance liquid chromatography(HPLC).Sample Preparation: Test solutions were prepared at specified concentrations from four accurately weighed extracts. Each solution was centrifuged at 10,000 rpm for 10 minutes, and the supernatant was filtered through a 0.22 μm microporous membrane prior to HPLC injection.

Chromatographic Conditions: The analysis was performed on a Symmetry C18 column (4.6 mm × 250 mm, 5 µm) maintained at 35°C. An isocratic mobile phase of methanol and 0.1% phosphoric acid aqueous solution (7:93, v/v) was delivered at a flow rate of 1.0 mL/min. The injection volume was 20 µL, and detection was carried out at a wavelength of 270 nm.
