# Supplementary material for: Co-fermentation of Rosa roxburghii Tratt pulp by Saccharomyces cerevisiae and Bacillus subtilis alleviates diarrhea-predominant irritable bowel syndrome in mice by reshaping gut microbiota and host metabolism
Source: Front Nutr. 2026 Jun 5;13:1854409. doi: 10.3389/fnut.2026.1854409 (PMC13279404; doi:10.3389/fnut.2026.1854409)
Supplement: Supplementary file 2 [file Supplementary_file_2.docx]

Supplementary Material S2

S2.Tentative Identification of compounds in *Rosa roxburghii* Tratt pulp Extracte before and after fermentation





**Supplementary Figure2.1** Base peak chromatogram of RRTP aqueous extract in positive and negative ion modes prior to fermentation.

**Supplementary Table 2.1** Tentative identification of potential bioactive constituents in the aqueous extract of RRTP prior to fermentation by UHPLC-ESI-Q-Exactive Plus Orbitrap-MS.

| NO. | Identification Name | Molecular  Formular | Error  (ppm) | m/z | RT (min) | MS2 | Reference  Ion |
| --- | --- | --- | --- | --- | --- | --- | --- |
| 1 | α-Linolenic acid | C_18_H_30_O | -0.17 | 279.23181 | 34.446 | 109.10150,95.08599,81.07033 | [M+H]+1 |
| 2 | Stearamide | C_18_H_37_NO | -0.07 | 284.29477 | 37.721 | 88.07616 | [M+H]+1 |
| 3 | Oleamide | C_18_H_35_NO | -0.53 | 282.27899 | 32.527 | 265.25290,247.24156,97.10164,69.07040,57.07051 | [M+H]+1 |
| 4 | Hexadecanamide | C_16_H_33_NO | -0.77 | 256.2633 | 34.964 | 102.09177,88.07617 | [M+H]+1 |
| 5 | Erucamide | C_22_H_43_NO | 0.07 | 338.34177 | 44.51 | 321.31619,303.30423,83.08585,57.07064 | [M+H]+1 |
| 6 | Dodecanedioic acid | C_12_H_22_O_4_ | -1.2 | 229.14426 | 12.068 | 229.14444,211.13354 | [M-H]-1 |
| 7 | Corchorifatty acid F | C_18_H_32_O_5_ | 1.25 | 327.21811 | 16.784 | 291.19678 | [M-H]-1 |
| 8 | Kojic acid | C_6_H_6_O_4_ | 2.23 | 143.03416 | 1.6 | 143.03413,125.02372,83.01334 | [M+H]+1 |
| 9 | Isocitric acid | C_6_H_8_O_7_ | -3.56 | 191.01904 | 1.23 | 191.05556,111.00762,87.00747,85.02820 | [M-H]-1 |
| 10 | D-(-)-Quinic acid | C_7_H_12_O_6_ | -4.44 | 191.05513 | 1.216 | 127.03926,87.00747,85.02820 | [M-H]-1 |
| 11 | 5-Hydroxymethyl-2-furaldehyde | C_6_H_6_O_3_ | 2.32 | 109.02875 | 3.013 | 109.02872,81.03401 | [M+H-H_2_O]+1 |
| 12 | Nicotinic acid | C_6_H_5_NO_2_ | 1.32 | 124.03947 | 1.047 | 124.03936,96.04472,80.04991 | [M+H]+1 |
| 13 | Oleanolic acid | C_30_H_48_O_3_ | 0.32 | 439.35721 | 35.39 | 439.35742,393.35077,203.17993 | [M+H-H_2_O]+1 |
| 14 | Arjungenin | C_30_H_48_O_6_ | 1.8 | 503.33881 | 23.546 | 503.33887,459.35263 | [M-H]-1 |
| 15 | 18-β-Glycyrrhetinic acid | C_30_H_46_O_4_ | 1.12 | 469.33301 | 29.704 | 469.33249,451.30483 | [M-H]-1 |
| 16 | (±)-Abscisic acid | C_15_H_20_O_4_ | 0.09 | 263.12909 | 13.162 | 219.13878,204.11526 | [M-H]-1 |
| 17 | [Arjunic acid](https://www.chemsrc.com/en/cas/31298-06-3_315294.html) | C_30_H_48_O_5_ | 0.82 | 487.34344 | 23.846 | 469.33365 | [M-H]-1 |
| 18 | α,α-Trehalose | C_12_H_22_O_11_ | 0.41 | 377.08597 | 0.841 | 179.05527,161.04488,71.01248,59.01251 | [M+Cl]-1 |
| 19 | Quercetin-3β-D-  glucoside | C_21_H_20_O_12_ | 1.1 | 463.08893 | 10.601 | 463.08939,301.03583,300.028802,271.02533,243.03006 | [M-H]-1 |
| 20 | Adenosine | C_10_H_13_N_5_O_4_ | 0.36 | 268.10413 | 1.376 | 268.10338,136.06186 | [M+H]+1 |
| 21 | Rutin | C_27_H_30_O_16_ | 2.42 | 609.14758 | 10.397 | 300.02811,271.02518,255.03052 | [M-H]-1 |
| 22 | Quercetin | C_15_H_10_O_7_ | -0.61 | 303.04974 | 10.599 | 303.04895,229.04945,153.01828 | [M+H]+1 |
| 23 | Naringeninchalcone | C_15_H_12_O_5_ | -1.37 | 273.07538 | 10.351 | 273.07541,153.01814,147.04385,119.04925 | [M+H]+1 |
| 24 | Kaempferol | C_15_H_10_O_6_ | -1.58 | 287.05457 | 11.758 | 213.05440,165.01811,153.01807,121.02848,68.99761 | [M+H]+1 |
| 25 | Phloroglucinol | C_6_H_6_O_3_ | 1.37 | 127.03912 | 0.905 | 127.03902,81.07033 | [M+H]+1 |
| 26 | Ellagic acid | C_14_H_6_O_8_ | -2.27 | 303.01285 | 8.583 | 257.00800,229.01312,201.01833,173.02339 | [M+H]+1 |
| 27 | Catechin | C_15_H_14_O_6_ | 2.37 | 289.07245 | 6.836 | 245.08195,203.07143,123.04408,109.02830 | [M-H]-1 |
| 28 | 4-Methylumbelliferone | C_10_H_8_O_3_ | -0.31 | 177.05457 | 9.3 | 131.04878 | [M+H]+1 |
| 29 | DL-Arginine | C_6_H_14_N_4_O_2_ | -0.12 | 175.11894 | 0.734 | 175.11891,130.09747,116.07077,70.06566 | [M+H]+1 |
| 30 | L-Pyroglutamic acid | C_5_H_7_NO_3_ | 1.52 | 130.05005 | 1.272 | 84.04485,56.05005 | [M+H]+1 |
| 31 | Gallic acid | C_7_H_6_O_5_ | -4.91 | 169.01334 | 2.07 | 125.02329,107.01254,79.01756,69.03324 | [M-H]-1 |





**Supplementary Figure 2.2** Base peak chromatograms of RRTP aqueous extract after fermentation in positive and negative ion modes.

**Supplementary Table 2.2** Tentative identification of potential bioactive constituents in the aqueous extract of fermented RRTP by UHPLC-ESI-Q-Exactive Plus Orbitrap-MS

| NO. | Identification Name | MolecularFormular | Error  (ppm) | m/z | RT (min) | MS2 | Reference Ion |
| --- | --- | --- | --- | --- | --- | --- | --- |
| 1 | α-Linolenic acid | C_18_H_30_O_2_ | -0.93 | 279.2316 | 34.451 | 109.10136,81.07030,95.08595,67.05479 | [M+H]+1 |
| 2 | Stearamide | C_18_H_37_NO | -0.31 | 284.2947 | 38.961 | 88.07626 | [M+H]+1 |
| 3 | Oleamide | C_18_H_35_NO | -0.49 | 282.279 | 32.535 | 265.25290,247.24156,135.11655 | [M+H]+1 |
| 4 | Hexadecanamide | C_16_H_33_NO | -0.25 | 256.26343 | 34.973 | 102.09177,88.07613 | [M+H]+1 |
| 5 | Erucamide | C_22_H_43_NO | 0.5 | 338.34191 | 39.087 | 321.31619,303.30536,83.08605,69307050,57.07052 | [M+H]+1 |
| 6 | Dodecanedioic acid | C_12_H_22_O_4_ | -0.93 | 229.14432 | 12.051 | 229.14447,211.13373,167.14326 | [M-H]-1 |
| 7 | Corchorifatty acid F | C_18_H_32_O_5_ | 0.79 | 327.21796 | 16.89 | 291.19681 | [M-H]-1 |
| 8 | Azelaic acid | C_9_H_16_O_4_ | -3.66 | 187.09689 | 12.087 | 187.09695,169.08598,125.09612 | [M-H]-1 |
| 9 | Kojic acid | C_6_H_6_O_4_ | -0.09 | 143.03389 | 1.451 | 143.03381,113.02348,97.02877,83.01320,69.03403 | [M+H]+1 |
| 10 | Isocitric acid | C_6_H_8_O_7_ | -3.41 | 191.01906 | 1.207 | 191.05559,111.00768,87.00749,85.02818 | [M-H]-1 |
| 11 | D-(-)-Quinic acid | C_7_H_12_O_6_ | -1.07 | 175.06015 | 1.508 | 127.03947,93.03330,87.00753,85.02824 | [M+H-H_2_O]+1 |
| 12 | Nicotinic acid | C_6_H_5_NO_2_ | 1.44 | 124.03948 | 1.039 | 124.03937,96.04472,80.04492 | [M+H]+1 |
| 13 | Oleanolic acid | C_30_H_48_O_3_ | 0.54 | 439.3573 | 35.4 | 439.35724,393.35120,203.17921 | [M+H-H_2_O]+1 |
| 14 | Carvone | C_10_H_14_O | -3.47 | 151.11122 | 10.608 | 123.0799 | [M+H]+1 |
| 15 | Arjungenin | C_30_H_48_O_6_ | 1.38 | 503.33865 | 21.688 | 503.33926,485.32751 | [M-H]-1 |
| 16 | 18-β-Glycyrrhetinic acid | C_30_H_46_O_4_ | 1.24 | 469.33313 | 29.707 | 469.33371,451.32352,409.31366 | [M-H]-1 |
| 17 | (±)-Abscisic acid | C_15_H_20_O_4_ | -0.77 | 263.12915 | 13.132 | 219.13890,204.11528 | [M-H]-1 |
| 18 | [Arjunic acid](https://www.chemsrc.com/en/cas/31298-06-3_315294.html) | C_30_H_48_O_5_ | 0.39 | 487.34344 | 23.853 | 469.33347 | [M-H]-1 |
| 19 | α,α-Trehalose | C_12_H_22_O_11_ | 0.04 | 381.07913 | 0.84 | 179.05544,161.04469,101.03407,89.02312,71.01251.59.01254 | [M+K]+1 |
| 20 | Quercetin-3β-D-  glucoside | C_21_H_20_O_12_ | 1.9 | 463.08908 | 10.569 | 463.08954,301.03583,300.02805,271.02530,243.02998 | [M-H]-1 |
| 21 | Gluconic acid | C_6_H_12_O_7_ | -3.58 | 195.05032 | 0.827 | 195.05055,159.02907,129.01828,75.00743 | [M-H]-1 |
| 22 | Astragalin | C_21_H_20_O_11_ | 1.33 | 447.09402 | 11.742 | 284.03336,227.03499 | [M-H]-1 |
| 23 | Trigonelline | C_7_H_7_NO_2_ | -0.06 | 138.05495 | 0.857 | 110.06023,94.06548 | [M+H]+1 |
| 24 | Guanine | C_5_H_5_N_5_O | -0.1 | 152.05667 | 1.337 | 152.05659,135.03009,110.03508,55.02967 | [M+H]+1 |
| 25 | Choline | C_5_H_13_NO | 2.92 | 104.10729 | 0.785 | 104.10725,60.08139,58.06576 | [M+H]+1 |
| 26 | Adenosine | C_10_H_13_N_5_O_4_ | -0.56 | 268.10388 | 1.262 | 268.10376,136.06175,119.03542 | [M+H]+1 |
| 27 | Taxifolin | C_15_H_12_O_7_ | -4.22 | 305.06427 | 7.105 | 153.0178,149.02275 | [M+H]+1 |
| 28 | Rutin | C_27_H_30_O_16_ | 2.72 | 609.14777 | 10.372 | 300.02798,271.02539,255.03032,153.01785 | [M-H]-1 |
| 29 | Quercetin | C_15_H_10_O_7_ | -4.25 | 303.04865 | 10.564 | 303.04803,229.04874,165.01776,153.01778 | [M+H]+1 |
| 30 | Naringeninchalcone | C_15_H_12_O_5_ | -0.69 | 273.07556 | 10.304 | 273.07544,153.01814,147.04395.119.04929, | [M+H]+1 |
| 31 | Kaempferol | C_15_H_10_O_6_ | 0.13 | 287.05502 | 11.736 | 16,501,836,153.02 | [M+H]+1 |
| 32 | Epicatechin | C_15_H_14_O_6_ | -1.5 | 291.08572 | 5.548 | 147.04407,139.03880,123.04404 | [M+H]+1 |
| 33 | Pyrogallol | C_6_H_6_O_3_ | 3.94 | 127.03947 | 1.771 | 110.03207,109.02889 | [M+H]+1 |
| 34 | Protocatechuic acid | C_7_H_6_O_4_ | 2.5 | 155.03426 | 3.696 | 153.01785,137.02321,131.97466,81.03391 | [M+H]+1 |
| 35 | Phloroglucinol | C_6_H_6_O_3_ | 2.13 | 127.03915 | 2.675 | 127.03922,81.03406 | [M+H]+1 |
| 36 | Ellagic acid | C_14_H_6_O_8_ | 0.92 | 303.01385 | 8.489 | 257.00827,229.01344,201.01851,173.02356 | [M+H]+1 |
| 37 | Catechin | C_15_H_14_O_6_ | 2.26 | 289.07242 | 6.59 | 245.08218,203.07213,137.02333,125.02335,123.04405,109.02837 | [M-H]-1 |
| 38 | Butylparaben | C_11_H_14_O_3_ | -2.63 | 193.08639 | 19.557 | 193.08644,61.98694 | [M-H]-1 |
| 39 | 4-Methylumbelliferone | C_10_H_8_O_3_ | -4.56 | 177.05382 | 9.191 | 131.04861 | [M+H]+1 |
| 40 | 4-Coumaric acid | C_9_H_8_O_3_ | -2.76 | 147.04361 | 6.847 | 147.04359,119.04899 | [M+H-H_2_O]+1 |
| 41 | L-Phenylalanine | C_9_H_11_NO_2_ | 2.05 | 166.08659 | 2.252 | 131.04922,120.08112,103.05452 | [M+H]+1 |
| 42 | DL-Arginine | C_6_H_14_N_4_O_2_ | -0.11 | 175.11894 | 0.738 | 175.11935,158.08124,116.07070,70.06564 | [M+H]+1 |
| 43 | Gallic acid | C_7_H_6_O_5_ | -4.37 | 169.01338 | 1.775 | 125.02335,107.01261,79.01766,69.03326 | [M-H]-1 |





**Supplementary Figure 2.3** Base peak chromatograms of RRTP ethanol extract prior to fermentation in both positive and negative ion modes.

**Supplementary Table 2.3** Tentative identification of potential bioactive constituents in ethanol extracts of RRTP prior to fermentation by UHPLC-ESI-Q-Exactive Plus Orbitrap-MS

| NO. | Identification  Name | Molecular Formular | Error  (ppm) | m/z | RT (min) | MS2 | Reference Ion |
| --- | --- | --- | --- | --- | --- | --- | --- |
| 1 | Vanillin | C_8_H_8_O_3_ | -1.13 | 153.0546 | 8.89 | 153.05458,125.05981,  111.04429,93.03388,  65.03917 | [M+H]+1 |
| 2 | Pyrogallol | C_6_H_6_O_3_ | 0.95 | 127.03907 | 0.919 | 109.0286 | [M+H]+1 |
| 3 | Emodin | C_15_H_10_O_5_ | -3.77 | 271.05908 | 8.934 | 271.05923,229.04861 | [M+H]+1 |
| 4 | Ellagic acid | C_14_H_6_O_8_ | 0.73 | 303.01379 | 8.5 | 257.00827,229.01353,  201.01854,173.02354 | [M+H]+1 |
| 5 | Catechin | C_15_H_14_O_6_ | 0.31 | 289.0722 | 6.583 | 245.08205,203.07100,  151.03911,123.04408,  109.02834 | [M-H]-1 |
| 6 | 4-Methylumbelliferone | C_10_H_8_O_3_ | -1.61 | 177.05434 | 9.197 | 175.02432,131.04904,  93.07017 | [M+H]+1 |
| 7 | Rutin | C_27_H_30_O_16_ | 2.42 | 609.14758 | 10.376 | 300.02792,271.02512,  255.03001,151.00267 | [M-H]-1 |
| 8 | Quercetin | C_15_H_10_O_7_ | -1.93 | 303.04935 | 10.577 | 303.04953,229.04927,  153.01814, | [M+H]+1 |
| 9 | Naringeninchalcone | C_15_H_12_O_5_ | -1.7 | 273.07529 | 10.315 | 273.07468,153.01775,  147.04359,119.04898 | [M+H]+1 |
| 10 | Naringenin | C_15_H_12_O_5_ | -1.06 | 273.0752 | 11.888 | 271.06198,151.00284,  119.04913,107.01274 | [M+H]+1 |
| 11 | Kaempferol | C_15_H_10_O_6_ | -1.55 | 287.05453 | 11.746 | 213.05392,165.01794,  153.01781,121.02832 | [M+H]+1 |
| 12 | Isorhamnetin | C_16_H_12_O_7_ | -1.34 | 317.06516 | 12.011 | 302.04138,285.03897,  153.01811 | [M+H]+1 |
| 13 | Benzophenone | C_13_H_10_O | 0.43 | 183.08052 | 23.069 | 183.08044,105.03381 | [M+H]+1 |
| 14 | Choline | C_5_H_13_NO | 2.78 | 104.10728 | 0.794 | 104.10719,60.08136 | [M+H]+1 |
| 15 | Adenosine | C_10_H_13_N_5_O_4_ | -1.58 | 268.10361 | 1.246 | 268.08044,136.06158 | [M+H]+1 |
| 16 | α,α-Trehalose | C_12_H_22_O_11_ | 0.32 | 377.08597 | 0.857 | 179.05539,161.04459,  89.02315,71.01250,  59.01249 | [M+Cl]-1 |
| 17 | Quercetin-3β-D-  glucoside | C_21_H_20_O_12_ | 0.69 | 463.08893 | 10.575 | 301.03577,300.02795,  271.02527 | [M-H]-1 |
| 18 | Astragalin | C_21_H_20_O_11_ | 0.52 | 447.0939 | 11.749 | 284.03311,227.03490, | [M-H]-1 |
| 19 | 1. Hydroxymethyl-2- 2. furaldehyde | C_6_H_6_O_3_ | 1.43 | 109.02864 | 2.664 | 109.02881,81.03407 | [M+H-H2O]+1 |
| 20 | Oleanolic acid | C_30_H_48_O_3_ | 0.39 | 439.35718 | 35.921 | 455.35413 | [M+H-H2O]+1 |
| 21 | Arjungenin | C_30_H_48_O_6_ | 1.82 | 503.33875 | 21.707 | 503.33887,485.32767 | [M-H]-1 |
| 22 | (±)-Abscisic acid | C_15_H_20_O_4_ | 0.28 | 263.12918 | 13.143 | 219.13882 | [M-H]-1 |
| 23 | Arjunic acid | C_30_H_48_O_5_ | 1.79 | 487.34378 | 24.025 | 469.33331 | [M-H]-1 |
| 24 | Nicotinic acid | C_6_H_5_NO_2_ | 1.07 | 124.03944 | 1.051 | 124.03934,96.04470,  80.04989 | [M+H]+1 |
| 25 | Kojic acid | C_6_H_6_O_4_ | -0.51 | 143.03381 | 4.165 | 143.03397,125.02354 | [M+H]+1 |
| 26 | Isocitric acid | C_6_H_8_O_7_ | -3.56 | 191.01904 | 1.223 | 191.05554,111.00755,  85.02816 | [M-H]-1 |
| 27 | Dodecanedioic acid | C_12_H_22_O_4_ | -0.73 | 229.14436 | 12.065 | 229.14445,211.13350,  167.14342 | [M-H]-1 |
| 28 | D-(-)-Quinic acid | C_7_H_12_O_6_ | -4.28 | 191.05527 | 0.89 | 93.03335,85.02822 | [M-H]-1 |
| 29 | Asiatic acid | C_30_H_48_O_5_ | 2.12 | 487.34393 | 25.335 | 488.34723 | [M-H]-1 |
| 30 | Ascorbic acid | C_6_H_8_O_6_ | -4.25 | 175.02406 | 5.846 | 115.00252,87.00748 | [M-H]-1 |
| 31 | 3,4-Dihydroxybenzaldehyde | C_7_H_6_O_3_ | -0.88 | 139.03885 | 6.582 | 121.02839,111.04420 | [M+H]+1 |
| 32 | 18-β-Glycyrrhetinic acid | C_30_H_46_O_4_ | 1.06 | 453.33673 | 29.724 | 469.33316,451.32269,  409.31006 | [M+H-H2O]+1 |
| 33 | Stearamide | C_18_H_37_NO | 0.14 | 284.29483 | 38.972 | 57.07058 | [M+H]+1 |
| 34 | Oleamide | C_18_H_35_NO | -0.5 | 282.279 | 32.83 | 69.07040,57.07051 | [M+H]+1 |
| 35 | Hexadecanamide | C_16_H_33_NO | 0.27 | 256.26356 | 34.983 | 102.09810,88.07619 | [M+H]+1 |
| 36 | Erucamide | C_22_H_43_NO | 0.07 | 338.34177 | 44.52 | 321.31619,83.08585,  57.07064 | [M+H]+1 |
| 37 | Corchorifatty acid F | C_18_H_32_O_5_ | 1.25 | 327.21811 | 16.797 | 291.15757 | [M-H]-1 |
| 38 | 1-Linoleoyl glycerol | C_21_H_38_O_4_ | 0.09 | 337.27374 | 34.326 | 285.01010,91.05795 | [M+H-H2O]+1 |
| 39 | luteolin | C_15_H_10_O_6_ | -1.35 | 285.04062 | 16.905 | 285.04092,151.00235 | [M-H]-1 |
| 40 | Gallic acid | C_7_H_6_O_5_ | -4.64 | 169.01346 | 2.73 | 125.02332,107.01260,  69.03324 | [M-H]-1 |





**Supplementary Figure 2.4** Base peak chromatograms of RRTP ethanol extract after fermentation in both positive and negative ion modes.

**Supplementary Table 2.4** Tentative identification of potential bioactive constituents in ethanol extracts of fermented RRTP by UHPLC-ESI-Q-Exactive Plus Orbitrap-MS

| NO. | Identification  Name | Molecular Formular | error (ppm) | m/z | RT  (min) | MS2 | Reference Ion |
| --- | --- | --- | --- | --- | --- | --- | --- |
| 1 | α-Linolenic acid | C_18_H_30_O_2_ | -0.99 | 279.23157 | 34.481 | 109.1014,95.0859,  81.07034,67.05482 | [M+H]+1 |
| 2 | Stearamide | C_18_H_37_NO | 0.26 | 284.29487 | 38.988 | 88.07620,57.07058 | [M+H]+1 |
| 3 | Oleamide | C1_8_H_35_NO | -0.16 | 282.27909 | 32.85 | 265.25290,247.24156,  69.07040,57.07051 | [M+H]+1 |
| 4 | Hexadecanamide | C_16_H_33_NO | -0.43 | 256.26338 | 35.004 | 102.09180,88.07619 | [M+H]+1 |
| 5 | Erucamide | C_22_H_43_NO | 0.07 | 338.34177 | 44.514 | 321.31619,303.30423,  83.08585 | [M+H]+1 |
| 6 | Dodecyl sulfate | C_12_H_26_O_4_S | 1.55 | 265.14832 | 28.325 | 96.95885 | [M-H]-1 |
| 7 | Corchorifatty acid F | C_18_H_32_O_5_ | 0.79 | 327.21796 | 16.914 | 291.19815 | [M-H]-1 |
| 8 | 1-Linoleoyl glycerol | C_21_H_38_O_4_ | -2.44 | 337.27289 | 34.345 | 285.01050,91.05795 | [M+H-H_2_O]+1 |
| 9 | Nicotinic acid | C_6_H_5_NO_2_ | 0.94 | 124.03942 | 1.213 | 124.03932,96.04470,  80.04990 | [M+H]+1 |
| 10 | Kojic acid | C_6_H_6_O_4_ | -2.61 | 187.02419 | 1.434 | 143.03377,125.02334 | [M+FA-H]-1 |
| 11 | Dodecanedioic acid | C_12_H_22_O_4_ | -0.87 | 229.14433 | 12.075 | 229.14444,211.13359,  167.14296 | [M-H]-1 |
| 12 | D-(-)-Quinic acid | C_7_H_12_O_6_ | -2.73 | 191.05559 | 1.717 | 127.03902,93.03312 | [M-H]-1 |
| 13 | Citric acid | C_6_H_8_O_7_ | -3.29 | 191.01907 | 1.088 | 173.04498,111.00755 | [M-H]-1 |
| 14 | Azelaic acid | C_9_H_16_O_4_ | -3.66 | 187.09689 | 12.114 | 187.09686,125.09611,  97.06467 | [M-H]-1 |
| 15 | 4-Guanidinobutyric acid | C_5_H_11_N_3_O_2_ | -0.08 | 146.09239 | 1.117 | 146.09222,104.07103,  87.04446 | [M+H]+1 |
| 16 | 4-Coumaric acid | C_9_H_8_O_3_ | -1.62 | 147.04378 | 6.864 | 147.04382,119.04918 | [M+H-H_2_O]+1 |
| 17 | 18-β-Glycyrrhetinic acid | C_30_H_46_O_4_ | -1.16 | 453.33533 | 29.744 | 469.33304,451.32272,  409.31351 | [M+H-H_2_O]+1 |
| 18 | 16-Hydroxyhexadecanoic acid | C_16_H_32_O_3_ | 1.82 | 271.22836 | 34.095 | 271.22836,225.22235 | [M-H]-1 |
| 19 | 12-Oxo  phytodienoic acid | C_18_H_28_O_3_ | 0.23 | 275.20065 | 18.509 | 69.0704 | [M+H-H_2_O]+1 |
| 20 | Arjunic acid | C_30_H_48_O_5_ | -1 | 471.34637 | 16.65 | 469.33163,441.33868 | [M+H-H_2_O]+1 |
| 21 | Ursolic acid | C_30_H_48_O_3_ | 1.2 | 457.36786 | 35.441 | 457.36398,411.36285,  231.21243,203.17854 | [M+H]+1 |
| 22 | Oleanolic acid | C_30_H_48_O_3_ | -0.03 | 457.36758 | 25.509 | 439.35913,411.36234,  393.35251 | [M+H]+1 |
| 23 | Asiatic acid | C_30_H_48_O_5_ | 2.12 | 487.34393 | 25.347 | 488.34482 | [M-H]-1 |
| 24 | Arjungenin | C_30_H_48_O_6_ | 1.69 | 503.33868 | 21.718 | 503.3389,485.32855 | [M-H]-1 |
| 25 | (±)-Abscisic acid | C_15_H_20_O_4_ | 0 | 265.14322 | 13.157 | 265.14319,247.13220,  229.12219 | [M+H]+1 |
| 26 | Sucrose | C_12_H_22_O_11_ | 0.28 | 377.08603 | 0.854 | 341.10971,59.01249 | [M+Cl]-1 |
| 27 | Quercetin-3β-D-  glucoside | C_21_H_20_O_12_ | 1.05 | 463.08905 | 10.754 | 463.08917,301.03583,  300.02802,271.02542,  203.17947 | [M-H]-1 |
| 28 | 1. Hydroxymethyl-2- 2. furaldehyde | C_6_H_6_O_3_ | 2.08 | 109.02873 | 2.705 | 109.02860,81.03391 | [M+H-H_2_O]+1 |
| 29 | Trigonelline | C_7_H_7_NO_2_ | -0.08 | 138.05493 | 0.871 | 138.05487,110.06025,  94.06549, | [M+H]+1 |
| 30 | Guanosine | C_10_H_13_N_5_O_5_ | 1.69 | 282.08487 | 1.353 | 150.04118,133.01451 | [M-H]-1 |
| 31 | Guanine | C_5_H_5_N_5_O | -0.5 | 152.05661 | 1.352 | 152.05661,135.03009,  110.03509 | [M+H]+1 |
| 32 | Choline | C_5_H_13_NO | 2.48 | 104.10725 | 0.799 | 104.10722,60.08137 | [M+H]+1 |
| 33 | Betaine | C_5_H_11_NO_2_ | 1.59 | 118.08644 | 0.853 | 118.08636,59.077356,  58.06575 | [M+H]+1 |
| 34 | Adenosine | C_10_H_13_N_5_O_4_ | -0.78 | 268.10382 | 1.243 | 268.08014,136.06171 | [M+H]+1 |
| 35 | Taxifolin | C_15_H_12_O_7_ | -2.42 | 305.06461 | 7.119 | 259.05969,153.01794,  149.02295 | [M+H]+1 |
| 36 | Rutin | C_27_H_30_O_16_ | 2.62 | 609.14771 | 10.388 | 301.03583,271.02524,  255.03018,163.00246,  107.01241 | [M-H]-1 |
| 37 | Quercetin | C_15_H_10_O_7_ | -1.62 | 303.04944 | 10.756 | 300.04858,229.04895,  165.01794,153.01793 | [M+H]+1 |
| 38 | Naringeninchalcone | C_15_H_12_O_5_ | 0.06 | 273.07547 | 16.318 | 273.07544,153.01814,  147.04396,119.04927 | [M+H]+1 |
| 39 | Kaempferol | C_15_H_10_O_6_ | -3.28 | 287.05402 | 11.761 | 213.05394,165.01779,  153.01779,121.02827 | [M+H]+1 |
| 40 | Isorhamnetin | C_16_H_12_O_7_ | -2.79 | 317.0647 | 12.025 | 302.04083,274.04608,  229.04909,153.01788 | [M+H]+1 |
| 41 | Butylparaben | C_11_H_14_O_3_ | -2.7 | 193.08638 | 19.579 | 193.08644,61.98694 | [M-H]-1 |
| 42 | Benzophenone | C_13_H_10_O | 0.29 | 183.08049 | 23.079 | 183.08044,105.03381 | [M+H]+1 |
| 43 | 4-Methylumbelliferone | C_10_H_8_O_3_ | -1.53 | 177.05435 | 9.204 | 131.04904 | [M+H]+1 |
| 44 | Vanillin | C_8_H_8_O_3_ | 0.54 | 153.0547 | 2.989 | 153.05478,125.05991,  111.04435 | [M+H]+1 |
| 45 | Pyrogallol | C_6_H_6_O_3_ | 1.27 | 127.03913 | 1.789 | 110.03192,109.02869 | [M+H]+1 |
| 46 | Phloroglucinol | C_6_H_6_O_3_ | 0.5 | 127.03907 | 0.924 | 127.03898,81.03391 | [M+H]+1 |
| 47 | Ellagic acid | C_14_H_6_O_8_ | -1.69 | 303.01303 | 8.505 | 257.00751,229.01271,  201.01793,173.02301 | [M+H]+1 |
| 48 | Catechin | C_15_H_14_O_6_ | 1.95 | 289.07233 | 6.607 | 245.08214,203.07095,  179.03445,137.02335, | [M-H]-1 |
| 49 | 3,4-Dihydroxybenzaldehyde | C_7_H_6_O_3_ | -1.77 | 139.03873 | 6.61 | 121.02840,111.04410 | [M+H]+1 |
| 50 | luteolin | C_15_H_10_O_6_ | -1.04 | 285.04065 | 16.908 | 285.04089,151.00252 | [M-H]-1 |
| 51 | Gallic acid | C_7_H_6_O_5_ | -4.55 | 169.01347 | 2.806 | 125.02328,107.01309,  97.02823 | [M-H]-1 |
